# Light modulation ameliorates expression of circadian genes and disease progression in spinal muscular atrophy mice

**DOI:** 10.1093/hmg/ddy249

**Published:** 2018-08-14

**Authors:** Lisa M Walter, Christiane E Koch, Corinne A Betts, Nina Ahlskog, Katharina E Meijboom, Tirsa L E van Westering, Gareth Hazell, Amarjit Bhomra, Peter Claus, Henrik Oster, Matthew J A Wood, Melissa Bowerman

**Affiliations:** 1 Institute of Neuroanatomy and Cell Biology, Hannover Medical School, Hannover, Germany; 2 Institute of Neurobiology, University of Lübeck, Lübeck, Germany; 3 Department of Physiology, Anatomy and Genetics, University of Oxford, Oxford, UK; 4 Current affiliations: School of Medicine, Keele University, Staffordshire, UK; 5 Institute for Science and Technology in Medicine, Stoke-on-Trent, UK; 6 Wolfson Centre for Inherited Neuromuscular Disease, RJAH Orthopaedic Hospital, Oswestry, UK

## Abstract

Physiology and behaviour are critically dependent on circadian regulation via a core set of clock genes, dysregulation of which leads to metabolic and sleep disturbances. Metabolic and sleep perturbations occur in spinal muscular atrophy (SMA), a neuromuscular disorder caused by loss of the survival motor neuron (SMN) protein and characterized by motor neuron loss and muscle atrophy. We therefore investigated the expression of circadian rhythm genes in various metabolic tissues and spinal cord of the Taiwanese *Smn^−/−^;SMN2* SMA animal model. We demonstrate a dysregulated expression of the core clock genes (*clock*, *ARNTL/Bmal1*, *Cry1/2*, *Per1/2*) and clock output genes (*Nr1d1* and *Dbp*) in SMA tissues during disease progression. We also uncover an age- and tissue-dependent diurnal expression of the *Smn* gene. Importantly, we observe molecular and phenotypic corrections in SMA mice following direct light modulation. Our study identifies a key relationship between an SMA pathology and peripheral core clock gene dysregulation, highlights the influence of SMN on peripheral circadian regulation and metabolism and has significant implications for the development of peripheral therapeutic approaches and clinical care management of SMA patients.

## Introduction

Circadian regulation is critical for many biological processes, disruption of which causes diverse metabolic disorders (1). The suprachiasmatic nucleus (SCN), whose major external cue is light, is the central pacemaker and synchronizes peripheral circadian oscillators (2), all of which are sustained in a cell-autonomous manner by core clock genes and their protein products (3). Circadian locomotor output cycles kaput (CLOCK) and aryl hydrocarbon receptor nuclear translocator-like protein 1 (ARNTL or BMAL1) activate transcription of *Period* (*PER1, 2* and *3)* and *Cryptochrome* (*CRY1* and *2)* genes, the protein products of which negatively regulate CLOCK-ARNTL/BMAL1 expression (4,5). Numerous clock gene mutants develop metabolic and muscle defects (6), highlighting the link between circadian regulation and metabolic homeostasis.

Spinal muscular atrophy (SMA) is a fatal autosomal recessive disorder in children characterized by spinal motor neuron degeneration and progressive muscle weakness (7,8). The disease-determining *survival motor neuron 1* (*SMN1)* gene located on chromosome 5 is deleted or mutated on both alleles in SMA patients. While a highly homologous gene, *SMN2,* exists on the same chromosome, it undergoes alternative exon 7 splicing (9) to yield a truncated SMN2 protein (10) unable to compensate for loss of the full-length product (9). The SMN protein plays a role in small nuclear ribonucleoprotein (snRNP) assembly, pre-mRNA splicing and actin dynamics as well as in the regulation of axonal mRNA localization (11–13). Nonetheless, it is still not known why loss of the SMN protein leads to the specific pathophysiology of SMA.

Although motor neurons are amongst the most severely afflicted cells in SMA, tissues outside the central nervous system (CNS) including heart (14,15), pancreas (16), liver (17), skeletal muscle (18,19), spleen (20–22), thymus (22), the gastrointestinal tract (23) and lung (24) are also affected. Interestingly, many of these organs have metabolic functions and display intrinsic circadian gene expression (25). Various studies have reported significant metabolic abnormalities in SMA animal models and patients such as altered fatty acid metabolism, hyperlipidemia, hyperglycemia, hyperglucagonemia increased hepatic insulin sensitivity, glucose intolerance, development of diabetes mellitus, diabetic ketoacidosis as well as glucose and insulin aberrations (16,26–33). In addition to such metabolic perturbations, SMA patients display abnormal sleep (34), including altered sleep microstructure (35), nocturnal hypoxaemia and hypercapnia (36). These disruptions could point to a perturbed circadian phenotype. Indeed, we have recently demonstrated an aberrant diurnal regulation of the glucocorticoid-Krüppel-like factor 15-branched chain amino acid (GC-KLF15-BCAA) metabolic pathway in serum, skeletal muscle, spinal cord (SC), liver, heart, white adipose tissue (WAT) and brown adipose tissue (BAT) of the Taiwanese *Smn^−/−^;SMN2* mice (37).

We thus evaluated the hypothesis of a generalized circadian dysregulation in the severe Taiwanese *Smn^−/−^;SMN2* SMA mouse model (38) and uncover for the first time that the *Smn* gene displays a diurnal regulation in various metabolic tissues during early development. Further, we demonstrate disruption of the diurnal expression of core clock genes and clock output genes in metabolic tissues during SMA disease progression. Importantly, we find that controlled light (CL) exposure restores the expression of circadian rhythm genes and attenuates the severe SMA phenotype with beneficial effects on survival and weight. Combined, our results highlight a dysregulation of circadian rhythm genes in SMA metabolic tissues and suggest a functional relationship between the *SMN* gene, peripheral clock regulation and metabolic homeostasis.

## Results

### Altered diurnal expression of core clock genes in SMA metabolic tissues and SC during disease progression

To investigate the expression of the core clock genes in different SMA tissues, we used the severe Taiwanese *Smn^−/−^_;_SMN2* SMA mouse model (38). Upon pairing, breeding pairs were continuously entrained to a 12-h light:12-h dark cycle (LD12:12). Metabolic tissues regulated by a peripheral clock (*Tibialis anterior* (TA), liver, heart, WAT, BAT) and SC were harvested from *Smn^−/−^;SMN2* mice and *Smn^+/−^;SMN2* healthy littermates every 4 hrs (Zeitgeber time, ZT) over a 24-h time course (ZT0 = 8 am, ZT1 = 9 am, ZT5 = 1 pm, ZT9 = 5 pm, ZT13 = 9 pm, ZT17 = 1 am, ZT21 = 5 am). The expression profiles of *Per1/2, Cry1/2, Clock and Bmal1* were determined from pre-symptomatic postnatal day (P) 2 and post-symptomatic P7 mice (19).

**Figure 1:**
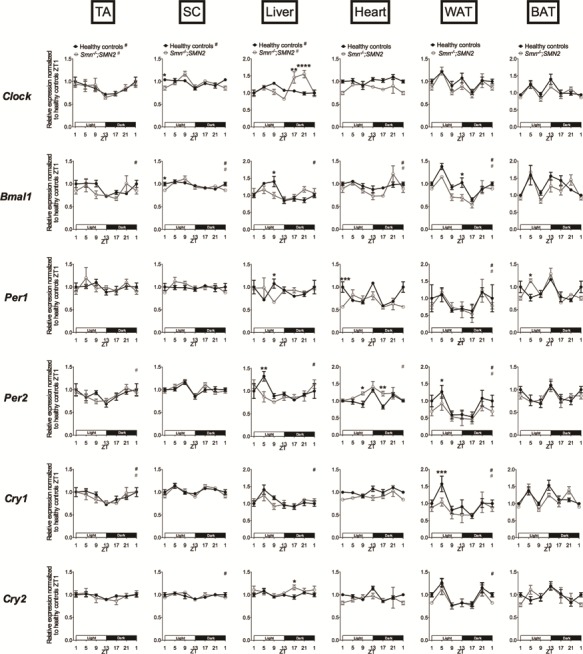
Dysregulation of diurnal expression of core clock genes in several tissues of pre-symptomatic SMA mice. Diurnal expression of core clock genes (*Clock*, *Bmal1*, *Per1*, *Per2*, *Cry1* and *Cry2*) in TA, WAT, BAT, liver, heart and SC of postnatal day (P) 2 *Smn^−/−^;SMN2* mice compared to healthy littermates. Data are mean ± SEM, n = 3–5 mice per ZT, ^*^P-value < 0.05, ^**^P-value < 0.01, ^***^P-value < 0.001, ^****^P-value < 0.0001 (two-way ANOVA), # indicates circadian rhythmicity. ZT1 data are duplicated.

In pre-symptomatic P2 *Smn^−/−^;SMN2* animals, we detected a tissue-specific disruption of the diurnal expression of core clock genes compared to control littermates (Fig. 1). We observed changes in amplitude, whereby SMA and healthy littermates followed the same oscillation pattern but with differential expression at specific ZTs (SC: *Bmal1* (ZT1); liver: *Bmal1* (ZT9); heart: *Per2* (ZT9, ZT17); WAT: *Bmal1* (ZT13), *Per2* (ZT5), *Cry1* (ZT5)). We also identified changes in phase, whereby SMA and healthy littermates displayed distinct oscillation patterns, whether they cycle in either experimental groups or only in one (SC: *Clock*; liver: *Clock*, *Bmal1*, *Per2*; heart: *Bmal1*). As circadian patterns are still in development and not yet fully established at this early time point (39,40), many clock genes do not yet display a diurnal oscillatory pattern in P2 tissues from either SMA mice or healthy littermates. However, we still detect significant differences in clock gene expression levels in these instances (liver: *Per1, Cry2*; heart: *Per1*; BAT: *Per1*).

**Figure 2:**
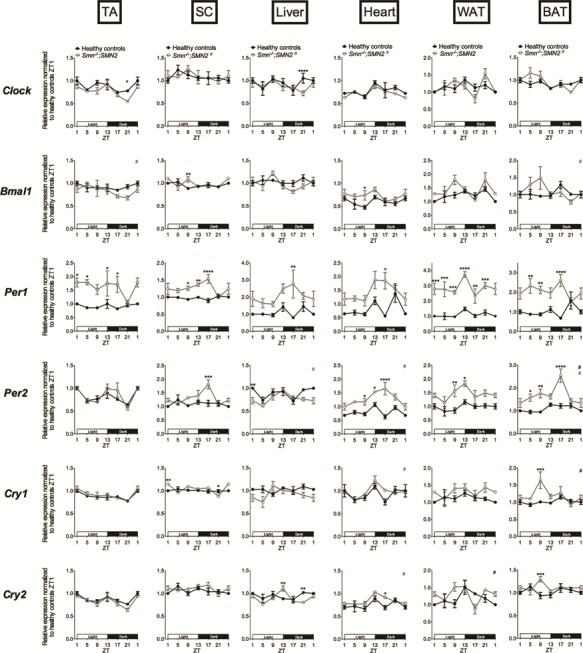
Dysregulation of diurnal expression of core clock genes in several tissues of symptomatic SMA mice. Diurnal expression of core clock genes (*Clock*, *Bmal1*, *Per1*, *Per2*, *Cry1* and *Cry2*) in TA, WAT, BAT, liver, heart and SC of postnatal day (P) 7 *Smn^−/−^;SMN2* mice compared to healthy controls. Data are mean ± SEM, n = 3–4 mice per ZT, ^*^P-value < 0.05, ^**^P-value < 0.01, ^***^P-value < 0.001, ^****^P-value < 0.0001 (two-way ANOVA), # indicates circadian rhythmicity. ZT1 data are duplicated.

We next performed a similar analysis in symptomatic P7 *Smn^−/−^;SMN2* mice and healthy littermates of the same age. Here, we found an even more prominent perturbation of clock gene expression in SMA tissues (Fig. 2), suggesting that diurnal expression defects in peripheral clocks increase with disease progression. Indeed, we observed several changes in amplitude, whereby SMA mice and healthy littermates followed the same oscillation patterns but with differential expression at specific ZTs (TA: *Clock* (ZT21); liver: *Per1* (ZT17), *Per2* (ZT1); heart: *Bmal1* (ZT9), *Per1* (ZT17), *Per2* (ZT9, ZT13, ZT17), *Cry2* (ZT17); WAT: *Per1* (all ZTs), *Per2* (ZT9, ZT13)). We also identified numerous changes in phase, whereby SMA mice and healthy littermates displayed distinct oscillation patterns, whether they cycle in either experimental groups or only in one (SC: *Clock;* liver: *Clock*; WAT: *Cry2*; BAT: *Clock*, *Bmal1*, *Per2*, *Cry1*). Similar to our P2 analysis, we find that many clock genes do not yet display a diurnal oscillatory pattern in P7 tissues from either SMA mice or healthy littermates. Nevertheless, differential expression between experimental groups is still observed, further highlighting dysregulated expression of core clock genes in SMA animals (TA: *Per1*; SC: *Bmal1*, *Per1*, *Per2*, *Cry1*; liver: *Cry2*; BAT: *Per1*, *Cry2*).

Our analysis of diurnal expression of core clock genes thus reveals a systemic dysregulation in pre- and post-symptomatic SMA tissues, which appears to worsen during disease progression.

### Altered diurnal expression of clock output genes in SMA metabolic tissues and SC during disease progression

To determine if the perturbations observed with the core clock genes were reflected downstream in clock output genes, we evaluated the diurnal expression of *Nr1d1* (also known as *Rev-erb-α*) and *Dbp*. *Nr1d1* is a direct transcriptional target of the CLOCK/BMAL1 complex and modulates the interaction of circadian rhythms and metabolism (41). *Dbp* is also a transcriptional target of CLOCK/BMAL1, subsequently binding the *Per1* promoter, thus promoting its cyclic behaviour (42).

**Figure 3:**
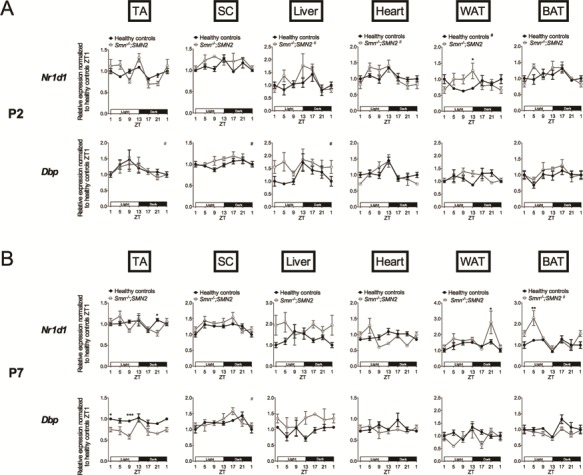
Dysregulation of diurnal expression of clock output genes in several tissues of pre-symptomatic and symptomatic SMA mice. Diurnal expression of clock output genes (*Nr1d1*, *Dbp*,) in TA, WAT, BAT, liver, heart and SC of postnatal day (P) 2 (**A**) and P7 (**B**) *Smn^−/−^;SMN2* mice compared to healthy littermates. P2 data are mean ± SEM, n = 3–5 mice per ZT, P7 data are mean ± SEM, n = 3–4 per ZT, ^*^P-value < 0.05, ^**^P-value < 0.01, ^***^P-value < 0.001 (two-way ANOVA), # indicates circadian rhythmicity. ZT1 data are duplicated.

In P2 pre-symptomatic animals, we only observed phase dist-inctions between *Smn^−/−^;SMN2* animals and healthy littermates, whereby there was either a significant difference in expression level (*Nr1d1*: WAT, ZT13) or a diurnal oscillatory pattern in only one group (*Nr1d1*: liver, heart; *Dbp*: TA, SC, liver) (Fig. 3A).

In P7 symptomatic *Smn^−/−^;SMN2* mice, we identified amplitude alterations when compared to healthy littermates (TA: *Dbp* (ZT1, ZT9); WAT: *Nr1d1* (ZT21); BAT: *Nr1d1* (ZT5)) (Fig. 3B). We also detect changes in phase, whether via significant differential expression levels (*Nr1d1*: TA, ZT21) or via a diurnal oscillatory pattern being detected in only one experimental group (*Nr1d1*: SC) (Fig. 3B).

**Figure 4:**
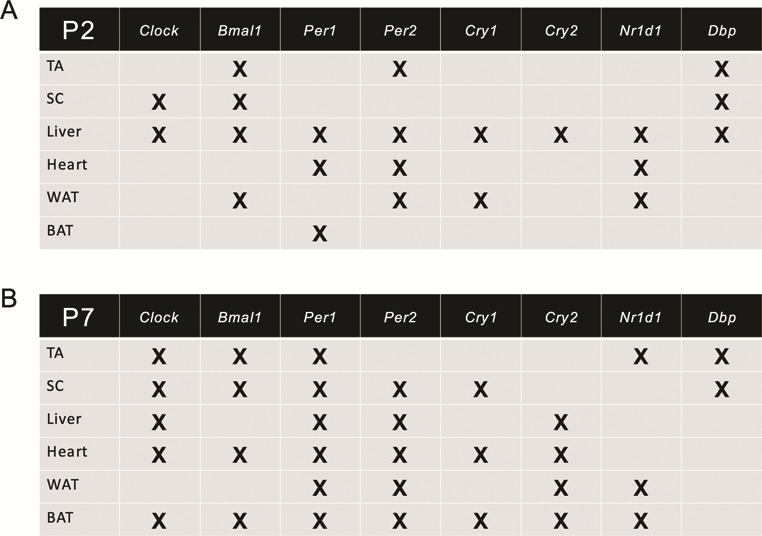
Summary of dysregulations of diurnal expression of clock and clock output genes in SMA tissues during disease progression. An X indicates either a change in phase, change in amplitude or differential expression of core clock and clock output genes in postnatal (P) 2 (**A**) and P7 (**B**) SMA mice compared to healthy littermates at one or more time points during a 24-h period.

Overall, our analysis of *Nr1d1* and *Dbp* expression reveals that in addition to the core clock genes, the diurnal expression of canonical clock output effectors is also perturbed in SMA tissues during disease progression (Fig. 4).

### Diurnal expression of myogenic regulatory factors is not impacted in muscle of **SMA mice**

In addition to the ubiquitously expressed core clock genes, muscle-specific myogenic regulatory factors such as MyoD and myogenin have also been reported as displaying a functional circadian expression profile in skeletal muscle of adult wild-type (WT) mice (43,44). As both MyoD and myogenin have been reported to be aberrantly expressed in Smn-depleted murine muscle tissue and cells (45,46), we assessed the diurnal expression of both genes in the TA of P2 and P7 *Smn^−/−^;SMN2* mice and healthy littermates.

**Figure 5:**
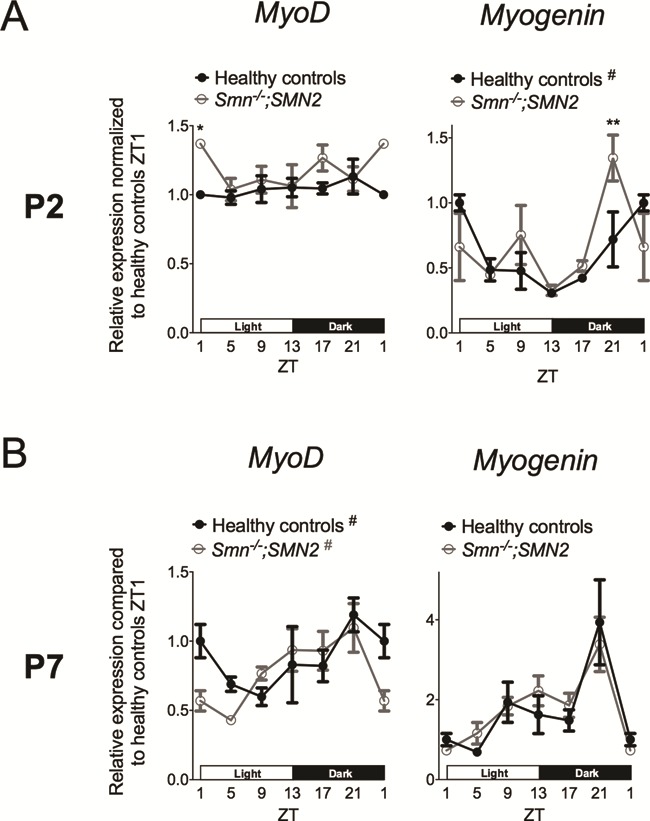
Diurnal expression of *MyoD* and *myogenin* is not significantly affected in skeletal muscle of SMA mice during disease progression. Diurnal expression of *MyoD* and *myogenin* in TA of postnatal day (P) 2 (**A**) and P7 (**B**) *Smn^−/−^;SMN2* mice compared to healthy littermates. P2 data are mean ± SEM, n = 3–6 mice per ZT, P7 data are mean ± SEM, n = 3–5 mice per ZT, ^*^P-value < 0.05, ^**^P-value < 0.01 (two-way ANOVA), # indicates circadian rhythmicity. ZT1 data are duplicated.

In P2 muscle, while *MyoD* did not cycle in either experimental group, we did detect a differential expression at ZT1, where *MyoD* levels were significantly greater in *Smn^−/−^;SMN2* mice (Fig. 5A). *Myogenin* displayed a diurnal expression profile in healthy littermates only, accompanied by a significant decreased expression at ZT21 in *Smn^−/−^;SMN2* mice (Fig. 5A).

At P7, a diurnal oscillatory pattern of *MyoD* was observed in both experimental groups while *myogenin* did not display a diurnal profile in either group (Fig. 5B). Furthermore, total expression levels between *Smn^−/−^;SMN2* mice and healthy littermates were similar for both genes at all ZTs (Fig. 5B).

Thus, our analysis reveals that whilst small differences are observed in P2 muscle between *Smn^−/−^;SMN2* mice and healthy littermates, SMA muscle pathology at P7 does not seem to impair the normal diurnal expression of *MyoD* and *myogenin*.

### The *Smn* gene displays an age- and tissue-dependent diurnal expression profile

**Figure 6:**
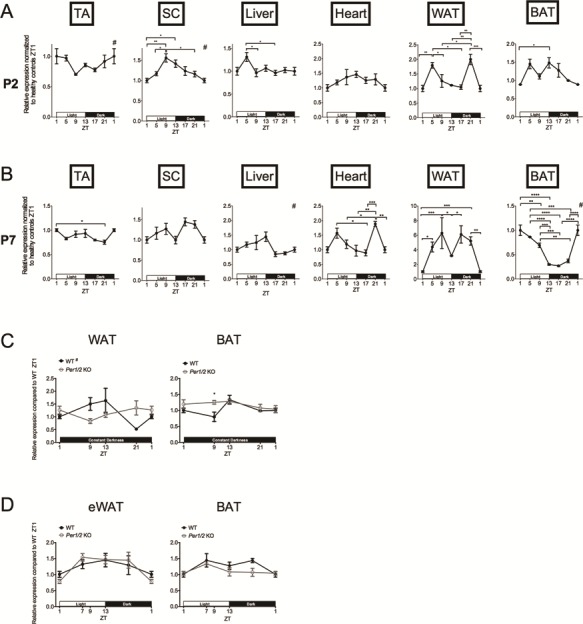
The *Smn* gene displays an age-, tissue- and light-dependent diurnal expression. Diurnal expression of *Smn* in TA, SC, liver, heart, WAT and BAT of postnatal day (P) 2 (**A**) and P7 (**B**) *Smn^+/−^;SMN2* mice healthy control mice. P2 data are mean ± SEM, n = 3–4 mice per ZT, P7 data are mean ± SEM, n = 3–4 mice per ZT, ^*^P-value < 0.05, ^**^P-value < 0.01, ^***^P-value < 0.001 (One-way ANOVA), # indicates circadian rhythmicity. ZT1 data is duplicated. (**C**) Diurnal expression of *Smn* in WAT and BAT from P7 WT and *Per1/2* mutants (KO) exposed to constant darkness. Data are mean ± SEM, n = 3–7 mice per ZT, ^*^P-value < 0.05 (two-way ANOVA), # indicates circadian rhythmicity. ZT1 data are duplicated. (**D**) Diurnal expression of *Smn* in epididymal eWAT and BAT from 3- to 4-month-old male WT and *Per1/2* mutants (KO). Data are mean ± SEM, n = 3 mice per ZTs. ZT1 data ar duplicated.

We next evaluated the diurnal expression of the disease-causing *Smn* gene in healthy littermates, which has never been assessed before. Surprisingly, we uncovered that *Smn* shows a 24-h differential expression pattern in P2 TA, liver, WAT and BAT (Fig. 6A). In P7 tissues, we found that while the expression patterns had somewhat changed between P2 and this later time point, the *Smn* gene presented a distinct 24-h differential expression pattern in TA, liver, heart, WAT and BAT (Fig. 6B).

As light is one of the most important external cues for the synchronization of internal peripheral clocks (47), we next determined if cycling of the *Smn* gene was dependent on light by investigating its expression in P7 tissues from WT animals that were exposed to constant darkness (D:D). As *Per1* and *Per2* were the clock genes whose expression levels were most significantly dysregulated in P7 SMA tissues (Fig. 2), we performed similar experiments with *Per1/2* double mutants (48). WAT and BAT were chosen for analysis as they displayed the greatest variations in *Smn*, *Per1* and *Per2* expression over a 24-h period. Interestingly, we found that the diurnal expression of *Smn* is preserved in WAT of P7 WT D:D animals (Fig. 6C), albeit with a different pattern than observed in mice in LD12:12 (Fig. 6B). In BAT, however, the cyclic expression of *Smn* is completely lost in P7 WT D:D mice (Fig. 6C). Furthermore, the pattern of *Smn* expression is different in *Per1/2* KO animals, reflected by a loss of cycling in WAT and a significantly greater expression in ZT9 BAT compared to WT animals (Fig. 6C). These results suggest that the diurnal expression of *Smn* in P7 adipose tissue may be partly regulated by light and intrinsic circadian regulators.

Finally, we investigated epididymal WAT (eWAT) and BAT from 3- to 4-month-old males to determine if cyclic expression of *Smn* is maintained in adulthood. We find that in adult mice, the *Smn* gene does not demonstrate a diurnal pattern (Fig. 6D). Furthermore, analysis of *Smn* expression in age- and gender-matched *Per1/2* double mutants showed a similar expression pattern (Fig. 6D), further supporting that *Smn* levels are not influenced by circadian regulation in adult adipose tissue.

Our analysis of diurnal expression of core clock genes, clock output genes and *Smn* in P2 and P7 SMA and control mice suggests that *SMN* loss is associated with dysregulation of peripheral clock machinery and that this may be influenced by the fact that *Smn* itself demonstrates a diurnal pattern in highly metabolic tissues. Interestingly, the diurnal expression of *Smn* appears to be limited to early time points, pointing to a developmental interaction between *Smn* and circadian rhythmicity that could significantly impact the establishment of regulatory functions in these tissues.

### A controlled-light environment improves phenotypic and molecular phenotypes of SMA mice

**Figure 7:**
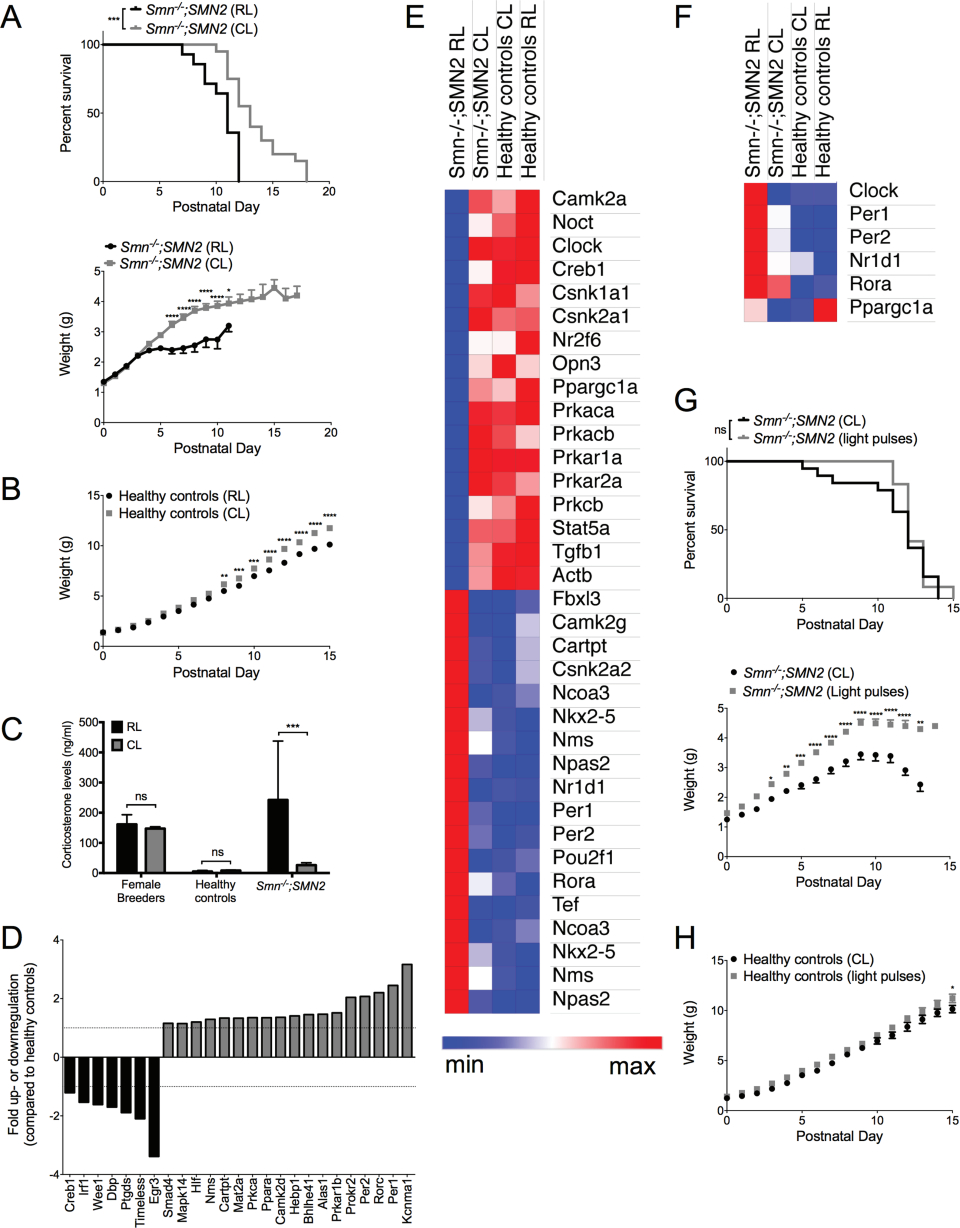
Light modulation impact molecular and phenotypic parameters in SMA mice. (**A**) Lifespan and weight development of *Smn^−/−^;SMN2* mice in CL (n = 20) and RL (n = 14) conditions. *P*-value = 0.0003 for Kaplan–Meier log-rank (Mantel-Cox); Data are mean ± SEM, ^*^P-value < 0.05, ^****^P-value < 0.0001 (two-way ANOVA). (**B**) Weight curves of healthy littermates in CL (n = 19) versus RL (n = 14). Data are mean ± SEM, ^**^P-value < 0.01, ^***^P-value < 0.001, ^****^P-value < 0.0001 (two-way ANOVA). (**C**) Corticosterone levels in serum of female breeders (n = 2 for RL and CL), healthy littermates (n = 7 for RL and CL) and *Smn^−/−^;SMN2 mice* (n = 11 for RL and 9 for CL). Data are mean ± SEM, ^***^P-value < 0.001, ns = not significant (two-way ANOVA). (**D**) Circadian rhythm genes dysregulated in ZT9 BAT from P7 *Smn^−/−^;SMN2* mice compared to healthy controls in CL conditions. (**E**) Heat map of circadian rhythm genes in ZT9 BAT of P7 *Smn^−/−^;SMN2* mice and healthy littermates from regular (RL) and CL shows genes dysregulated in *Smn^−/−^;SMN2* mice in RL and restored in *Smn^−/−^;SMN2* mice in CL. n = 4 for all experimental groups. **F.** Heat map of circadian rhythm genes in ZT17 BAT of P7 *Smn^−/−^;SMN2* mice and healthy littermates from RL and CL shows genes dysregulated in *Smn^−/−^;SMN2* mice in RL and restored in *Smn^−/−^;SMN2* mice in CL. n = 4 for all experimental groups. (**G**) Lifespan and weight development of *Smn^−/−^;SMN2* mice in CL (n = 19) versus light pulses (n = 12). ns = not significant for Kaplan–Meier log-rank (Mantel-Cox); data are mean ± SEM, ^*^P-value < 0.05, ^**^P-value < 0.01, ^***^P-value < 0.001, ^****^P-value < 0.0001 (two-way ANOVA). (**H**) Weight curves of healthy controls in CL (n = 16) and light pulses (n = 11). Data are mean ± SEM, ^*^P-value < 0.05 (two-way ANOVA).

Given the impact of light on diurnal cycling of the *Smn* gene, we investigated the effect of light modulation on the phenotype of SMA mice. Breeding trio A (2 females and 1 male) and ensuing litters were first placed in our typical animal holding room, defined as the regular light (RL) environment. While these rooms were on an LD12:12, cages were not directly under the light source and light:dark disruptions could occur due to comings and goings of personnel. Breeding trio A was subsequently transferred and entrained to an LD12:12 in circadian isolation cages (defined as CL condition), where the light-emitting diode (LED) light source is directly above the cages and the LD12:12 phases are unperturbed. From birth, all pups were weighed daily and monitored for survival. Interestingly, when compared to SMA mice in the RL, SMA mice from the CL displayed a significantly enhanced lifespan and a significantly increased weight gain (Fig. 7A). Healthy littermates also showed increased weights in the CL environment, but to a lesser extent than SMA mice (Fig. 7B). Exposing the intermediate *Smn^2B/−^* SMA mouse model (49,50) to the same experimental paradigm also resulted in a significant increase in weight of *Smn^2B/−^* mice in CL (Supplementary Material, Fig. S1A), without a significant impact on survival (Supplementary Material, Fig. S1B). Intrinsic differences between both models have previously been reported (37,45,51) and the differential effect of CL on survival may be due to ranges in disease severity and overall metabolic influence on disease progression.

In addition to light, differences in parameters such as temperature and noise/stress between RL and CL conditions may have contributed to the improved phenotype of the SMA mice from the circadian isolation cages, thus confounding the intrinsic circadian influence. We firstly found that the temperatures in each environment were not significantly different from each other (RL: min 20.63°C ± 0.25, max 22.2°C ± 0.79; CL: min 20.4°C ± 0, max 23.1°C ± 0). To evaluate stress due to noise and human presence, we assessed serum corticosterone levels, as this glucocorticoid is a known stress indicator in rodents (52). Breeding trio B (2 females and 1 male) and ensuing litters were first placed in the CL, followed by a transition to the RL. Serum from both females and P7 pups was collected at the same time of day (ZT5) as GCs display a diurnal expression pattern (53). Corticosterone levels were not significantly different between female breeders and healthy littermates from both RL and CL (Fig. 7C). However, we did detect significantly reduced levels of corticosterone in serum of SMA mice from CL compared to RL (Fig. 7C), further supporting the beneficial impact of light modulation on SMA pathophysiology. Our results therefore suggest that differences in temperature and stress levels between RL and CL are not key mediators of the phenotypic changes observed in SMA mice from the circadian isolation cages.

We next wanted to determine if adapting the light environment had an effect at a molecular level. Firstly, we determined if additional circadian rhythm genes are dysregulated in SMA tissues, beyond the previously investigated clock and clock output genes (Figs. 1–3). For this purpose, we used a commercially available mouse circadian rhythm qPCR array (SABiosciences), which looks at a subset of 84 genes known to display and/or regulate circadian rhythmicity. For proof-of-concept, we used P7 ZT9 BAT, a time point and tissue where expression of many clock genes was significantly dysregulated in SMA mice compared to healthy littermates (Fig. 2). We observed a large number of dysregulated circadian rhythm genes in P7 BAT of SMA animals compared to healthy littermates (Fig. 7D and Supplementary Material, Table S1), further supporting a systemic dysregulation of circadian regulation in SMA.

We then compared the expression of 84 circadian rhythm genes in P7 ZT9 BAT from mice exposed to RL and CL (Supplementary Material, Table S1). Heat map comparison demonstrates a large number of genes specifically dysregulated in RL SMA mice that are restored to normalized levels in CL SMA mice (Fig. 7E). Using the publicly available PANTHER software (54), we interrogated the list of differentially expressed genes from  Fig. 7E to determine specific protein classes, molecular functions, reactome pathways and GO biological processes (Table 1). Interestingly, in addition to the expected circadian rhythm-specific pathways, we identified functions such as glucocorticoid receptor signalling and regulation of transcription of RNA polymerase II, which we and others have demonstrated to be implicated in SMA pathogenesis (Fig. 7C) (37,55).

**Table 1:** Function, protein class, Reactome pathway and GO biological process complete of dysregulated genes in P7 BAT from RL SMA mice generated with Panther software

Genes dysregulated in SMA mice RL		Fold enrichment	*P*-value
Molecular Function	Protein kinase activity	8.16	1.40E-02
	Sequence-specific DNA binding transcription factor activity	4.64	1.51E-02
			
Protein class	Non-receptor serine/threonine protein kinase	11.78	1.24E-02
	Transcription factor	5.41	5.06E-04
Reactome pathway	RORA activates gene expression	>100	3.14E-09
	Degradation of GLI2 by the proteasome	>100	2.35E-02
	PPARA activates gene expression	>100	2.35E-02
	Rora activates gene expression	>100	8.55E-09
	Bmal1:Clock,Npas2 activates circadian gene expression	>100	9.35E-15
	WNT mediated activation of DVL	>100	3.35E-04
	PKA activation	>100	1.07E-05
	PKA activation in glucagon signalling	>100	1.38E-05
	DARPP-32 events	>100	2.21E-05
	Trafficking of AMPA receptors	69.76	1.73E-02
	Vasopressin regulates renal water homeostasis via Aquaporins	68.05	5.81E-04
	Post NMDA receptor activation events	63.41	2.29E-02
	Factors involved in megakaryocyte development and platelet producti	28.77	1.72E-02
	MAPK family signaling cascades	14.65	3.23E-02
	Developmental Biology	8.53	4.49E-03
			
GO biological process complete	Negative regulation of glucocorticoid receptor signaling pathway	>100	7.65E-04
	Development of secondary female sexual characteristics	>100	3.53E-03
	Protein-chromophore linkage	>100	4.69E-03
	Circadian regulation of gene expression	>100	9.62E-15
	Entrainment of circadian clock by photoperiod	>100	2.40E-02
	Circadian behavior	95.12	3.71E-02
	Peptidyl-serine phosphorylation	25.37	1.00E-03
	Positive regulation of sequence-specific DNA binding transcription fact	19.84	4.18E-03
	Cellular response to hormone stimulus	15.5	2.72E-04
	Morphogenesis of an epithelium	10.09	3.76E-02
	Embryonic morphogenesis	9.47	1.12E-02
	Cellular response to oxygen-containing compound	8.52	2.46E-02
	Regulation of secretion by cell	8.29	3.00E-02
	Positive regulation of transcription from RNA polymerase II promoter	8.06	7.77E-05
	Negative regulation of developmental process	7.21	2.07E-02
	Transcription, DNA-templated	4.8	6.82E-03
	Regulation of multicellular organismal process	4.16	1.35E-03
	Regulation of cellular protein metabolic process	4.16	3.42E-02
	Regulation of biological quality	3.41	2.07E-02

To evaluate if light modulation influences the diurnal expression of circadian rhythm genes, we compared the expression of a selected panel of genes in P7 ZT17 BAT from SMA mice and healthy littermates exposed to RL and CL. We chose to examine core clock genes (*Clock*, *Per1* and *Per2*), clock output genes (*Nr1d1* and *Rora*) and *Ppargc1α*, a transcriptional co-activator at the crossroads of circadian regulation and metabolism (56). Here, we found that CL did indeed restore the diurnal expression of *Clock*, *Per1*, *Per2* and *Nr1d1* genes in SMA mice (Fig. 7F). CL did not significantly impact *Rora* expression, while *Ppargc1a* levels seemed to be dependent on light environment irrespective of genotype (Fig. 7F). The considerable differences between RL and CL SMA BAT suggest that our reported differences in the diurnal expression profiles of clock and clock output genes between SMA mice and healthy littermates (Figs. 1–3) are most likely underestimated due to the experimental paradigm of synchronizing and regulating light exposure in the circadian isolation cages. Nevertheless, our results point to a beneficial influence of light modulation at a mechanistic and molecular level, particularly in restoring diurnal expression of circadian genes in BAT of symptomatic mice.

To further determine the role of light on the phenotypic changes in SMA mice, we transferred breeding trio C (2 females and 1 male) to LD12:12 in the CL and from birth, litters were exposed daily to light pulses during the dark cycle (1-h light pulses at ZTs 16 and 21). Breeding trio C and ensuing litters were subsequently maintained in the CL environment without light pulsing. From birth, all animals were weighed daily and monitored for survival. Disruption of the LD12:12 by light pulses had no effect on lifespan but led to enhanced weight gain in SMA animals compared to CL SMA mice (Fig. 7G). This effect was also observed in healthy littermates but to a much lesser extent (Fig. 7H). Several studies have reported an enhanced weight in adult rodents exposed to a disrupted LD cycle (57). As SMA mice display a more prominent weight gain in CL and light pulses conditions than healthy littermates, this suggests that SMN depletion increases sensitivity to changes in light modulation.

**Figure 8:**
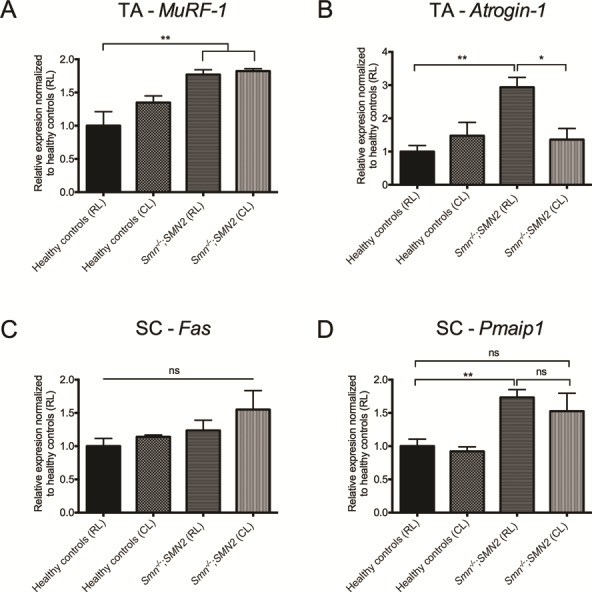
Light modulation impacts certain canonical pathological markers of SC and muscle pathology of SMA mice. TA and SC were harvested from postnatal (P) 7 *Smn^−/−^;SMN2* mice (n = 7 for RL and 3 for CL) and healthy littermates (n = 5 for RL and 6 for CL) in CL and RL conditions at ZT1 (9 am). Expression of *MuRF-1* (**A**) and *atrogin-1* (**B**) in TA of P7 *Smn^−/−^;SMN2* mice and healthy littermates from RL and CL. Expression of *Fas* (**C**) and *Pmaip1* (**D**) in SC of P7 *Smn^−/−^;SMN2* mice and healthy littermates from RL and CL. Data are mean ± SEM, ^*^P-value < 0.05, ^**^P-value < 0.01, ns = not significant (one-way ANOVA).

Finally, we set out to determine if light modulation influenced canonical pathologies in SMA SC and skeletal muscle, the two tissues that are the greatest contributors to SMA pathophysiology. The SC and TA of *Smn^−/−^;SMN2* mice and healthy littermates in both RL and CL were harvested at ZT1. We first compared the expression of *MuRF-1* and *atrogin-1*, well-characterized atrogenes (58), whose increase reflect the atrophy status of SMA muscle (37,51). While we observed that the CL environment did not influence the increased expression of *MuRF-1* in the TA of SMA mice (Fig. 8A), *atrogin-1* levels were significantly downregulated in muscle of CL SMA mice compared to RL SMA animals (Fig. 8B), indicating a reduced muscle atrophy. The expression of *Fas* and *Pmaip1* genes have previously been demonstrated as being aberrantly regulated in SMA SC as well as markers of improved pathology (59,60). Here, we observed that *Fas* levels were not influenced by the light environment, regardless of the experimental group (Fig. 8C), whilst *Pmaip1* levels displayed a non-significant trend towards a decreased expression in SC of CL SMA mice compared to RL SMA animals.

Combined, our results demonstrate that light modulation directly impacts several molecular and phenotypic parameters of SMA pathology and indicate that light as an external cue may have implications for overall metabolic health in SMA.

## Discussion

Our work defines for the first time a perturbed regulation of circadian rhythm genes in SMA CNS and peripheral tissues, which may contribute to and/or be a consequence of the many metabolic and sleep dysfunctions reported in SMA mice and patients (27,28,30–32). We also demonstrate that *Smn* itself displays a diurnal expression pattern in a tissue- and age-dependent manner. Finally, we establish that SMA mice are sensitive to light modulation, resulting in significant molecular and phenotypic changes relevant to SMA pathology. Our recent report of dysregulated circadian regulation of the GC-KLF15-BCAA pathway in SMA mice (37) further supports a functional relationship between this neuromuscular disease and systemic disruption of peripheral core clock and circadian rhythm genes. As circadian defects have been reported in models of other neuromuscular disorders such as collagen VI myopathy (61) and amyotrophic lateral sclerosis (62), our results may also reflect a more general relationship between circadian homeostasis and neuromuscular health.

Very little is known on the establishment of peripheral clocks during early development. In the first weeks after birth, neonatal rodents are photo-sensitive but not yet entrained to a light:dark cycle and rather respond to external cues such as the mother’s presence/absence and feeding pattern (39,40). While rhythmic expression of peripheral clocks is detected in perinatal rodents, its development is tissue-specific and can take up to 3 weeks to fully be established (39,40). Furthermore, intrinsic peripheral clocks can also be influenced by extrinsic metabolic pathways (63), many of which are perturbed in SMA (64). In our study, diurnal expression is thus evidently still in development and highly malleable, reflected by the organ- and age-dependent expression patterns of the core clock, clock output, *Smn, MyoD and myogenin* genes. Nevertheless, we identify several amplitude and phase changes between SMA mice and healthy littermates in P2 and P7 tissues. Interestingly, early developmental defects have previously been reported in heart (15), liver (17), skeletal muscle (19) and SC (65) of SMA mice, which, in some cases, may be linked to metabolic and circadian disturbances. To date, besides reports of increased fat mass in SMA patients that may contribute to morbidity (66,67), not much is known about the role of adipose tissue in SMA pathogenesis. Our demonstration of circadian dysregulation combined with the diurnal expression profile of the *Smn* gene in adipose tissue highlights the need for further investigations on the relationship between this tissue, SMN function and SMA pathology. Of particular interest is BAT, which not only originates from the same progenitor cells as skeletal muscle (68,69) but also communicates with several tissues and cells that are pathologically affected in SMA (70).

Dysregulation of clock and clock output genes has recently been reported in a mouse model of muscle denervation (71), a phenomenon that typifies SMA pathogenesis (72). Indeed, surgically inducing denervation of skeletal muscle in adult mice revealed a decreased expression of *Bmal1*, *Per1*, *Nr1d1* and *Dbp* and an increased expression of *Per2* (71). While the relationship between circadian rhythm genes and denervation in neonatal muscle requires further investigation, this study may point to the potential contribution of intrinsic SMA muscle pathology to the perturbed expression of clock and clock output genes within this tissue.

Our analysis of core clock genes specifically highlights the diurnal expression of *Per1* and *Per2* as being significantly increased in most tissues of symptomatic mice. As this generalized transcriptional upregulation is observed for only *Per1/2*, there may be a clock machinery-independent process induced by the SMA phenotype. Indeed, the expression of *Per1* and *Per2* is influenced by glucose (73) and GCs (74), steroid hormones that regulate glucose homeostasis (75). Interestingly, we and others, have reported several glucose metabolism (16,32,33,76,77) and GC perturbations in SMA mice (Fig. 7C) (37). Future endeavours investigating the relationship between glycaemia, glucocorticoids and *Per1/2* in SMA will therefore be of high interest.

Another key finding from our work is the observation that the *Smn* gene itself displays a diurnal expression pattern during early development in several tissues, which appears to be dependent on light and intrinsic circadian regulators. Previous studies have indicated the importance of SMN in early development (78), marked by high expression in fetal/perinatal tissues, followed thereafter by an age- and tissue-dependent decrease (79,80). This is reflected in our observed absence of diurnal *Smn* expression in eWAT and BAT of adult WT and *Per1/2* KO mice. Early loss of diurnal *Smn* expression in the SC and key metabolic tissues may therefore contribute to the sleep, metabolism and peripheral perturbations reported in SMA. Interestingly, the diurnal oscillation observed in P2 SC is lost at P7 and may be related to the previously mentioned importance of SMN in very early stages of development (78–80), particularly in this key pathological tissue. However, diurnal oscillation of *Smn*, albeit with distinct patterns, is maintained throughout development in WAT and BAT, further highlighting the requirement for an in-depth investigation of the potential function that SMN may play in adipose tissue.

The use of PCR arrays to investigate light-dependent molecular changes in BAT of SMA mice has brought forward genes previously implicated in SMA pathology. Indeed, one of the genes downregulated in SMA mice in RL is *Ppargc1a*, which plays a critical role in energy metabolism (81) and is decreased in skeletal muscle from SMA patients (82). Importantly, we demonstrate that expression of *Ppargc1a* is normalized in CL SMA mice. Moreover, *Prkacb* and *Prkaca*, the catalytic subunits of protein kinase A (PKA), also display a reduced expression in RL SMA BAT, which is normalized in CL SMA mice. Interestingly, PKA is associated with an enhanced SMN stability (83) and its upregulation in BAT of CL mice may partly explain the beneficial effect of light modulation on SMA animals. Finally, *Stat5a* also displays a decreased expression in BAT from RL SMA mice and is upregulated in CL. Increased activation of Stat5 has previously been demonstrated to induce SMN expression (84) and pharmacological compounds that increase Stat5 improve disease phenotypes in SMA cellular and animal models (85,86). Thus, the beneficial influence of light modulation on SMA pathology may be due to the cumulative effect of not one, but several molecular changes. Our proof-of-concept comparison of BAT from SMA and healthy animals in CL and RL therefore uncovers several light-dependent molecular changes highly relevant to SMA pathogenesis. Moreover, our investigation of diurnal dysregulation in BAT highlights the importance for future in-depth assessments of circadian regulation in SMA tissues.

Our work thus uncovers for the first time a systemic dysregulation of circadian rhythm genes in several SMA tissues, which may have a direct impact on certain aspects of disease pathophysiology. Our study provides further evidence that SMA is a multi-system disease (87) and highlights circadian modulators (whether pharmacological, dietary or environmental) as potential novel therapeutic endeavours for the overall clinical management of patients.

## Materials and Methods

### Animals

Experiments were carried out in the Biomedical Sciences Unit, University of Oxford according to procedures authorized by the UK Home Office (Animal Scientific Procedures Act 1986). All experiments were performed in the severe Taiwanese *Smn^−/−^;SMN2* SMA and the intermediate *Smn2B*/- mouse strains. Animals were housed either in the typical holding rooms of the animal facility (RL, LD12:12) or in circadian isolation cages (CL, LD12:12). For light pulse experiments, animals maintained in the circadian isolation cages were exposed to a 1-h light pulse during the dark phase at ZTs 16 and 21. For survival studies, animals were weighed daily and culled upon reaching their defined humane endpoint.

WT and *Per1/2* double mutant mice (on a C57BL/6 J background) were bred in the animal facility of the University of Lübeck. All mice were individually housed under standard laboratory conditions under LD12:12 conditions, a room temperature of 22 ± 2°C and a relative humidity of 55 ± 5% with *ad libitum* access to food (breeding chow 3.457 kcal/g; Ssniff, Soest, Germany) and water. For constant darkness experiments, breeding pairs and pups were kept in constant darkness for 2 days prior to harvest. The experimental protocol was approved by the Committee on Animal Health and Care of the Government of Schleswig-Holstein, Germany.

### qPCR

The liver, heart, WAT and BAT, SC and *TA* were harvested from P2 and P7 pups every 4 hrs over a 24-h period (ZT1 = 9 am, ZT5 = 1 pm, ZT9 = 5 pm, ZT13 = 9 pm, ZT17 = 1 am, ZT21 = 5 am). For single time point comparisons, tissues harvested at the same ZT were used. RNA was extracted with the RNeasy MiniKit (Qiagen) or the RNeasy Lipid Tissue MiniKit (Qiagen) for WAT and BAT. Reverse transcription was performed using the High-Capacity cDNA Reverse Transcription Kit (Applied Biosystems). qPCRs were performed using TaqMan Gene Expression Mastermix and Integrated DNA Technologies primers (see Supplementary Experimental Procedures). Housekeeping genes for each tissue was determined using the Mouse geNorm Kit and qbase+ software (Biogazelle) (see Supplementary Experimental Procedures).

Isolated WAT and BAT from WT and *Per1/2* KO animals were harvested in 4- or 6-h intervals over a 24-h period (ZT1, 7, 13, 19) and stored in RNAlater solution (Life Technologies) at 4°C overnight. RNA was extracted using TRIzol reagent (Life Technologies) followed by cDNA synthesis using the High Capacity cDNA Reverse Transcription Kit (Life Technologies). qPCRs were performed using TaqMan Gene Expression Mastermix and Integrated DNA Technologies primers (see Supplementary Experimental Procedures). Relative gene expression was quantified using the ΔΔ threshold cycle (Ct) method with adjustments for the amplification efficiencies of individual primer pairs. *GAPDH* was used as the reference gene.

### PCR arrays

RNA from ZT9 BAT of P7 animals was extracted using the RNeasy Lipid Tissue MiniKit. cDNA was made using RT^2^ First Strand Kit. qPCRs were performed using Mouse Circadian Rhythm PCR arrays (PAMM-153Z, SABiosciences). Data was analysed with the RT Profiler PCR Array Data Analysis version 3.5 and mRNA expression was normalized to the two most stably expressed genes between all samples. Heat maps reflect log2 fold changes and were generated with MORPHEUS software. PANTHER software (54) was used to analyse differentially expressed genes.

### Corticosterone ELISA

Analysis of corticosterone content in serum was performed with an ELISA kit (Abcam) following the manufacturer’s instructions. Serum samples were diluted 1:10 or 1:20.

### Statistical analysis

All statistical analyses were done with Graphpad Prism software. When appropriate, a Student’s unpaired two-tail *t*-test, a one-way ANOVA followed by a Tukey’s multiple comparison test or a two-way ANOVA followed by a Sidak’s multiple comparison test was used. Outliers were identified via the Grubbs’ test. For the Kaplan–Meier survival analysis, the log-rank test was used and survival curves were considered significantly different at *P*-value < 0.05. CircWave v1.4 was used to determine circadian rhythmicity.

## Supplementary Material

Supplementary Data
