# Supplementary material for: Light modulation ameliorates expression of circadian genes and disease progression in spinal muscular atrophy mice
Source: Hum Mol Genet. 2018 Aug 14;27(20):3582–97. doi: 10.1093/hmg/ddy249 (PMC6168969; doi:10.1093/hmg/ddy249)
Supplement: Supplementary Data [file ddy249_supp.zip › Karjosukarso et al - HMG-2018-D-00441_S4 Table.docx]

**S4 Table Genes identified in HEK293T ChIP which are differentially regulated by WT and MUT**

| **Symbol** | **Ensembl ID** | **FPKM** | | |
| --- | --- | --- | --- | --- |
|  |  | **NT** | **WT** | **MUT** |
| *HMOX1* | ENSG00000100292 | 30.30 | 47.97 | 25.07 |
| *HIST2H2AB* | ENSG00000184270 | 18.69 | 29.63 | 13.29 |
| *CDCA8* | ENSG00000134690 | 7.67 | 12.05 | 6.36 |
| *HIST1H2BL* | ENSG00000185130 | 59.24 | 103.33 | 31.13 |
| *HIST1H2BO* | ENSG00000274641 | 82.49 | 137.69 | 72.48 |
| *HIST1H3J* | ENSG00000197153 | 50.04 | 114.55 | 49.60 |
| *HIST1H4C* | ENSG00000197061 | 75.85 | 196.47 | 58.17 |
| *HIST1H4H* | ENSG00000158406 | 220.72 | 349.98 | 154.04 |
| *HIST1H2AH* | ENSG00000274997 | 43.53 | 96.02 | 40.45 |
| *HIST1H2BJ* | ENSG00000124635 | 137.43 | 346.42 | 127.87 |
| *WDR34* | ENSG00000119333 | 8.97 | 15.29 | 8.18 |
| *AURKB* | ENSG00000178999 | 9.62 | 17.80 | 9.65 |
| *GPC5* | ENSG00000179399 | 4.20 | 2.08 | 4.70 |
| *ITGA4* | ENSG00000115232 | 2.48 | 0.95 | 25.38 |
| *NEXN* | ENSG00000162614 | 22.82 | 5.78 | 13.77 |
