# Supplementary material for: Light modulation ameliorates expression of circadian genes and disease progression in spinal muscular atrophy mice
Source: Hum Mol Genet. 2018 Aug 14;27(20):3582–97. doi: 10.1093/hmg/ddy249 (PMC6168969; doi:10.1093/hmg/ddy249)
Supplement: Supplementary Data [file ddy249_supp.zip › Karjosukarso et al - HMG-2018-D-00441_S1 Table.docx]

**S1 Table Genes that are upregulated in WT, but not in MUT**

| **Ensembl ID** | **Symbol** |
| --- | --- |
| ENSG00000135451 | *TROAP* |
| ENSG00000197182 | *MIR4763* |
| ENSG00000127528 | *KLF2* |
| ENSG00000013810 | *TACC3* |
| ENSG00000233922 | *LOC105372840* |
| ENSG00000133800 | *LYVE1* |
| ENSG00000122952 | *ZWINT* |
| ENSG00000146670 | *CDCA5* |
| ENSG00000104147 | *OIP5* |
| ENSG00000162639 | *HENMT1* |
| ENSG00000163923 | *RPL39L* |
| ENSG00000197837 | *HIST4H4* |
| ENSG00000181885 | *CLDN7* |
| ENSG00000165071 | *TMEM71* |
| ENSG00000188643 | *S100A16* |
| ENSG00000101331 | *CCM2L* |
| ENSG00000161888 | *SPC24* |
| ENSG00000186281 | *GPAT2* |
| ENSG00000123977 | *DAW1* |
| ENSG00000204161 | *C10orf128* |
| ENSG00000130511 | *SSBP4* |
| ENSG00000140873 | *ADAMTS18* |
| ENSG00000090776 | *EFNB1* |
| ENSG00000175899 | *A2M* |
| ENSG00000163584 | *RPL22L1* |
| ENSG00000144554 | *FANCD2* |
| ENSG00000184232 | *OAF* |
| ENSG00000161513 | *FDXR* |
| ENSG00000168496 | *FEN1* |
| ENSG00000001561 | *ENPP4* |
| ENSG00000100304 | *TTLL12* |
| ENSG00000179604 | *CDC42EP4* |
| ENSG00000186994 | *KANK3* |
| ENSG00000164087 | *POC1A* |
| ENSG00000107719 | *PALD1* |
| ENSG00000161800 | *RACGAP1* |
| ENSG00000276043 | *UHRF1* |
| ENSG00000160447 | *PKN3* |
| ENSG00000124575 | *HIST1H1D* |
| ENSG00000184357 | *HIST1H1B* |
| ENSG00000277075 | *HIST1H2AE* |
| ENSG00000164032 | *H2AFZ* |
| ENSG00000276410 | *HIST1H2BB* |
| ENSG00000100292 | *HMOX1* |
| ENSG00000184270 | *HIST2H2AB* |
| ENSG00000079616 | *KIF22* |
| ENSG00000171345 | *KRT19* |
| ENSG00000197182 | *MIRLET7BHG* |
| ENSG00000197182 | *MIRLET7A3* |
| ENSG00000197182 | *MIRLET7B* |
| ENSG00000164109 | *MAD2L1* |
| ENSG00000073111 | *MCM2* |
| ENSG00000100297 | *MCM5* |
| ENSG00000166508 | *MCM7* |
| ENSG00000085840 | *ORC1* |
| ENSG00000131153 | *GINS2* |
| ENSG00000197594 | *ENPP1* |
| ENSG00000166851 | *PLK1* |
| ENSG00000102575 | *ACP5* |
| ENSG00000129195 | *FAM64A* |
| ENSG00000134690 | *CDCA8* |
| ENSG00000105011 | *ASF1B* |
| ENSG00000035499 | *DEPDC1B* |
| ENSG00000163638 | *ADAMTS9* |
| ENSG00000171848 | *RRM2* |
| ENSG00000127586 | *CHTF18* |
| ENSG00000186283 | *TOR3A* |
| ENSG00000110080 | *ST3GAL4* |
| ENSG00000183598 | *HIST2H3D* |
| ENSG00000252481 | *SCARNA13* |
| ENSG00000159167 | *STC1* |
| ENSG00000137310 | *TCF19* |
| ENSG00000270141 | *TERC* |
| ENSG00000167900 | *TK1* |
| ENSG00000184113 | *CLDN5* |
| ENSG00000176890 | *TYMS* |
| ENSG00000100162 | *CENPM* |
| ENSG00000131652 | *THOC6* |
| ENSG00000171241 | *SHCBP1* |
| ENSG00000276180 | *HIST1H4I* |
| ENSG00000093009 | *CDC45* |
| ENSG00000196747 | *HIST1H2AI* |
| ENSG00000276903 | *HIST1H2AL* |
| ENSG00000278463 | *HIST1H2AB* |
| ENSG00000185130 | *HIST1H2BL* |
| ENSG00000274290 | *HIST1H2BE* |
| ENSG00000275713 | *HIST1H2BH* |
| ENSG00000278588 | *HIST1H2BI* |
| ENSG00000111665 | *CDCA3* |
| ENSG00000274641 | *HIST1H2BO* |
| ENSG00000275714 | *HIST1H3A* |
| ENSG00000278272 | *HIST1H3C* |
| ENSG00000275379 | *HIST1H3I* |
| ENSG00000273983 | *HIST1H3G* |
| ENSG00000197153 | *HIST1H3J* |
| ENSG00000278828 | *HIST1H3H* |
| ENSG00000274267 | *HIST1H3B* |
| ENSG00000278637 | *HIST1H4A* |
| ENSG00000197061 | *HIST1H4C* |
| ENSG00000158406 | *HIST1H4H* |
| ENSG00000167747 | *C19orf48* |
| ENSG00000274997 | *HIST1H2AH* |
| ENSG00000118640 | *VAMP8* |
| ENSG00000157873 | *TNFRSF14* |
| ENSG00000128918 | *ALDH1A2* |
| ENSG00000134057 | *CCNB1* |
| ENSG00000277775 | *HIST1H3F* |
| ENSG00000196787 | *HIST1H2AG* |
| ENSG00000124635 | *HIST1H2BJ* |
| ENSG00000119333 | *WDR34* |
| ENSG00000162063 | *CCNF* |
| ENSG00000110711 | *AIP* |
| ENSG00000178999 | *AURKB* |
| ENSG00000071539 | *TRIP13* |
| ENSG00000158859 | *ADAMTS4* |
| ENSG00000100034 | *PPM1F* |
| ENSG00000135476 | *ESPL1* |
| ENSG00000007312 | *CD79B* |
| ENSG00000166803 | *KIAA0101* |
| ENSG00000117399 | *CDC20* |
| ENSG00000100918 | *REC8* |
| ENSG00000198327 | NA |
| ENSG00000124529 | NA |
| ENSG00000259001 | NA |
| ENSG00000267325 | NA |
