# Supplementary material for: Light modulation ameliorates expression of circadian genes and disease progression in spinal muscular atrophy mice
Source: Hum Mol Genet. 2018 Aug 14;27(20):3582–97. doi: 10.1093/hmg/ddy249 (PMC6168969; doi:10.1093/hmg/ddy249)
Supplement: Supplementary Data [file ddy249_supp.zip › Karjosukarso et al - HMG-2018-D-00441_S2 Table.docx]

**S2 Table Genes that are downregulated in WT, but not in MUT**

| **Ensembl ID** | **Symbol** |
| --- | --- |
| ENSG00000227124 | *ZNF717* |
| ENSG00000139187 | *KLRG1* |
| ENSG00000131773 | *KHDRBS3* |
| ENSG00000168386 | *FILIP1L* |
| ENSG00000180787 | *ZFP3* |
| ENSG00000144810 | *COL8A1* |
| ENSG00000111799 | *COL12A1* |
| ENSG00000164318 | *EGFLAM* |
| ENSG00000139971 | *C14orf37* |
| ENSG00000118523 | *CTGF* |
| ENSG00000143869 | *GDF7* |
| ENSG00000198947 | *DMD* |
| ENSG00000183044 | *ABAT* |
| ENSG00000078401 | *EDN1* |
| ENSG00000123572 | *NRK* |
| ENSG00000225383 | *SFTA1P* |
| ENSG00000148218 | *ALAD* |
| ENSG00000165092 | *ALDH1A1* |
| ENSG00000177409 | *SAMD9L* |
| ENSG00000083857 | *FAT1* |
| ENSG00000184254 | *ALDH1A3* |
| ENSG00000146555 | *SDK1* |
| ENSG00000189134 | *NKAPL* |
| ENSG00000179399 | *GPC5* |
| ENSG00000162631 | *NTNG1* |
| ENSG00000115414 | *FN1* |
| ENSG00000118407 | *FILIP1* |
| ENSG00000118946 | *PCDH17* |
| ENSG00000177990 | *DPY19L2* |
| ENSG00000091879 | *ANGPT2* |
| ENSG00000095951 | *HIVEP1* |
| ENSG00000180806 | *HOXC9* |
| ENSG00000147036 | *LANCL3* |
| ENSG00000140443 | *IGF1R* |
| ENSG00000115457 | *IGFBP2* |
| ENSG00000163453 | *IGFBP7* |
| ENSG00000122641 | *INHBA* |
| ENSG00000117595 | *IRF6* |
| ENSG00000115232 | *ITGA4* |
| ENSG00000206538 | *VGLL3* |
| ENSG00000156466 | *GDF6* |
| ENSG00000095015 | *MAP3K1* |
| ENSG00000170430 | *MGMT* |
| ENSG00000087245 | *MMP2* |
| ENSG00000198938 | *COX3* |
| ENSG00000198727 | *CYTB* |
| ENSG00000198840 | *ND3* |
| ENSG00000086991 | *NOX4* |
| ENSG00000124785 | *NRN1* |
| ENSG00000154678 | *PDE1C* |
| ENSG00000184588 | *PDE4B* |
| ENSG00000057294 | *PKP2* |
| ENSG00000128567 | *PODXL* |
| ENSG00000170891 | *CYTL1* |
| ENSG00000196368 | *NUDT11* |
| ENSG00000022556 | *NLRP2* |
| ENSG00000198185 | *ZNF334* |
| ENSG00000114698 | *PLSCR4* |
| ENSG00000134247 | *PTGFRN* |
| ENSG00000095303 | *PTGS1* |
| ENSG00000136383 | *ALPK3* |
| ENSG00000138771 | *SHROOM3* |
| ENSG00000105426 | *PTPRS* |
| ENSG00000148143 | *ZNF462* |
| ENSG00000169213 | *RAB3B* |
| ENSG00000114200 | *BCHE* |
| ENSG00000074527 | *NTN4* |
| ENSG00000126950 | *TMEM35A* |
| ENSG00000169439 | *SDC2* |
| ENSG00000154864 | *PIEZO2* |
| ENSG00000214944 | *ARHGEF28* |
| ENSG00000143429 | *LOC645166* |
| ENSG00000206432 | *TMEM200C* |
| ENSG00000196632 | *WNK3* |
| ENSG00000189223 | *PAX8-AS1* |
| ENSG00000144681 | *STAC* |
| ENSG00000130303 | *BST2* |
| ENSG00000092969 | *TGFB2* |
| ENSG00000162692 | *VCAM1* |
| ENSG00000147180 | *ZNF711* |
| ENSG00000075785 | *RAB7A* |
| ENSG00000129680 | *MAP7D3* |
| ENSG00000175471 | *MCTP1* |
| ENSG00000122786 | *CALD1* |
| ENSG00000138759 | *FRAS1* |
| ENSG00000118473 | *SGIP1* |
| ENSG00000184384 | *MAML2* |
| ENSG00000154556 | *SORBS2* |
| ENSG00000102802 | *MEDAG* |
| ENSG00000180543 | *TSPYL5* |
| ENSG00000138735 | *PDE5A* |
| ENSG00000133101 | *CCNA1* |
| ENSG00000003096 | *KLHL13* |
| ENSG00000162614 | *NEXN* |
| ENSG00000128487 | *SPECC1* |
| ENSG00000170160 | *CCDC144A* |
| ENSG00000184867 | *ARMCX2* |
| ENSG00000228495 | NA |
| ENSG00000205664 | NA |
| ENSG00000226702 | NA |
| ENSG00000237973 | NA |
| ENSG00000254635 | NA |
