# Supplementary material for: Light modulation ameliorates expression of circadian genes and disease progression in spinal muscular atrophy mice
Source: Hum Mol Genet. 2018 Aug 14;27(20):3582–97. doi: 10.1093/hmg/ddy249 (PMC6168969; doi:10.1093/hmg/ddy249)
Supplement: Supplementary Data [file ddy249_supp.zip › Karjosukarso et al - HMG-2018-D-00441_S3 Table.docx]

**S3 Table Top 1000 genes on principal component 1 and 2 axis**

| **Principal Component 1** | | **Principal Component 2** | |
| --- | --- | --- | --- |
| **Ensembl ID** | **Symbol** | **Ensembl ID** | **Symbol** |
| ENSG00000170558 | *CDH2* | ENSG00000170558 | *CDH2* |
| ENSG00000234420 | *ZNF37BP* | ENSG00000136560 | *TANK* |
| ENSG00000204767 | *FAM196B* | ENSG00000198865 | *CCDC152* |
| ENSG00000221676 | *RNU6ATAC* | ENSG00000255737 | *AGAP2-AS1* |
| ENSG00000234456 | *MAGI2-AS3* | ENSG00000205078 | *SYCE1L* |
| ENSG00000204520 | *MICA* | ENSG00000227124 | *ZNF717* |
| ENSG00000113810 | *SMC4* | ENSG00000221676 | *RNU6ATAC* |
| ENSG00000025434 | *NR1H3* | ENSG00000135451 | *TROAP* |
| ENSG00000254986 | *DPP3* | ENSG00000260260 | *SNHG19* |
| ENSG00000113163 | *COL4A3BP* | ENSG00000025434 | *NR1H3* |
| ENSG00000140937 | *CDH11* | ENSG00000230630 | *DNM3OS* |
| ENSG00000241553 | *ARPC4* | ENSG00000184281 | *TSSC4* |
| ENSG00000140391 | *TSPAN3* | ENSG00000140937 | *CDH11* |
| ENSG00000188042 | *ARL4C* | ENSG00000140945 | *CDH13* |
| ENSG00000122644 | *ARL4A* | ENSG00000127914 | *AKAP9* |
| ENSG00000073910 | *FRY* | ENSG00000245648 | *LOC101928100* |
| ENSG00000139679 | *LPAR6* | ENSG00000235448 | *LURAP1L-AS1* |
| ENSG00000245648 | *LOC101928100* | ENSG00000123136 | *DDX39A* |
| ENSG00000227517 | *LINC01483* | ENSG00000139187 | *KLRG1* |
| ENSG00000235448 | *LURAP1L-AS1* | ENSG00000128606 | *LRRC17* |
| ENSG00000064989 | *CALCRL* | ENSG00000070404 | *FSTL3* |
| ENSG00000105810 | *CDK6* | ENSG00000123080 | *CDKN2C* |
| ENSG00000105355 | *PLIN3* | ENSG00000100526 | *CDKN3* |
| ENSG00000136158 | *SPRY2* | ENSG00000264462 | *MIR3648-2* |
| ENSG00000124762 | *CDKN1A* | ENSG00000107833 | *NPM3* |
| ENSG00000131477 | *RAMP2* | ENSG00000127528 | *KLF2* |
| ENSG00000111276 | *CDKN1B* | ENSG00000164442 | *CITED2* |
| ENSG00000070404 | *FSTL3* | ENSG00000075213 | *SEMA3A* |
| ENSG00000103266 | *STUB1* | ENSG00000188229 | *TUBB4B* |
| ENSG00000147889 | *CDKN2A* | ENSG00000101335 | *MYL9* |
| ENSG00000147883 | *CDKN2B* | ENSG00000064225 | *ST3GAL6* |
| ENSG00000174130 | *TLR6* | ENSG00000080986 | *NDC80* |
| ENSG00000154258 | *ABCA9* | ENSG00000104324 | *CPQ* |
| ENSG00000264462 | *MIR3648-2* | ENSG00000134215 | *VAV3* |
| ENSG00000141338 | *ABCA8* | ENSG00000130204 | *TOMM40* |
| ENSG00000164442 | *CITED2* | ENSG00000013810 | *TACC3* |
| ENSG00000124406 | *ATP8A1* | ENSG00000250508 | *LOC105369364* |
| ENSG00000101335 | *MYL9* | ENSG00000233922 | *LOC105372840* |
| ENSG00000080986 | *NDC80* | ENSG00000105404 | *RABAC1* |
| ENSG00000104324 | *CPQ* | ENSG00000184990 | *SIVA1* |
| ENSG00000137693 | *YAP1* | ENSG00000086504 | *MRPL28* |
| ENSG00000134375 | *TIMM17A* | ENSG00000135624 | *CCT7* |
| ENSG00000106400 | *ZNHIT1* | ENSG00000115163 | *CENPA* |
| ENSG00000131236 | *CAP1* | ENSG00000183751 | *TBL3* |
| ENSG00000107175 | *CREB3* | ENSG00000076382 | *SPAG5* |
| ENSG00000108828 | *VAT1* | ENSG00000113356 | *POLR3G* |
| ENSG00000257219 | *LOC105369848* | ENSG00000133110 | *POSTN* |
| ENSG00000254486 | *LOC105376554* | ENSG00000111247 | *RAD51AP1* |
| ENSG00000136938 | *ANP32B* | ENSG00000131773 | *KHDRBS3* |
| ENSG00000151640 | *DPYSL4* | ENSG00000048740 | *CELF2* |
| ENSG00000184470 | *TXNRD2* | ENSG00000051341 | *POLQ* |
| ENSG00000138778 | *CENPE* | ENSG00000142731 | *PLK4* |
| ENSG00000113356 | *POLR3G* | ENSG00000132970 | *WASF3* |
| ENSG00000265972 | *TXNIP* | ENSG00000173083 | *HPSE* |
| ENSG00000117724 | *CENPF* | ENSG00000183207 | *RUVBL2* |
| ENSG00000133110 | *POSTN* | ENSG00000133800 | *LYVE1* |
| ENSG00000167642 | *SPINT2* | ENSG00000148671 | *ADIRF* |
| ENSG00000253522 | *MIR3142HG* | ENSG00000143554 | *SLC27A3* |
| ENSG00000077514 | *POLD3* | ENSG00000142945 | *KIF2C* |
| ENSG00000185737 | *NRG3* | ENSG00000175602 | *CCDC85B* |
| ENSG00000132970 | *WASF3* | ENSG00000099203 | *TMED1* |
| ENSG00000170271 | *FAXDC2* | ENSG00000123124 | *WWP1* |
| ENSG00000173083 | *HPSE* | ENSG00000175063 | *UBE2C* |
| ENSG00000125966 | *MMP24* | ENSG00000164283 | *ESM1* |
| ENSG00000104356 | *POP1* | ENSG00000070778 | *PTPN21* |
| ENSG00000213190 | *MLLT11* | ENSG00000138080 | *EMILIN1* |
| ENSG00000136026 | *CKAP4* | ENSG00000053254 | *FOXN3* |
| ENSG00000118508 | *RAB32* | ENSG00000122952 | *ZWINT* |
| ENSG00000139278 | *GLIPR1* | ENSG00000169306 | *IL1RAPL1* |
| ENSG00000243244 | *STON1* | ENSG00000173598 | *NUDT4* |
| ENSG00000080561 | *MID2* | ENSG00000168309 | *FAM107A* |
| ENSG00000119397 | *CNTRL* | ENSG00000170955 | *PRKCDBP* |
| ENSG00000091428 | *RAPGEF4* | ENSG00000168386 | *FILIP1L* |
| ENSG00000276023 | *DUSP14* | ENSG00000222041 | *CYTOR* |
| ENSG00000162616 | *DNAJB4* | ENSG00000135324 | *MRAP2* |
| ENSG00000164283 | *ESM1* | ENSG00000137491 | *SLCO2B1* |
| ENSG00000150687 | *PRSS23* | ENSG00000146670 | *CDCA5* |
| ENSG00000138738 | *PRDM5* | ENSG00000104147 | *OIP5* |
| ENSG00000138080 | *EMILIN1* | ENSG00000138435 | *CHRNA1* |
| ENSG00000026950 | *BTN3A1* | ENSG00000247596 | *TWF2* |
| ENSG00000136504 | *KAT7* | ENSG00000162639 | *HENMT1* |
| ENSG00000164985 | *PSIP1* | ENSG00000207445 | *SNORD15B* |
| ENSG00000160326 | *SLC2A6* | ENSG00000164796 | *CSMD3* |
| ENSG00000090376 | *IRAK3* | ENSG00000156804 | *FBXO32* |
| ENSG00000139629 | *GALNT6* | ENSG00000157657 | *ZNF618* |
| ENSG00000166073 | *GPR176* | ENSG00000144741 | *SLC25A26* |
| ENSG00000168386 | *FILIP1L* | ENSG00000164932 | *CTHRC1* |
| ENSG00000222041 | *CYTOR* | ENSG00000150551 | *LYPD1* |
| ENSG00000135324 | *MRAP2* | ENSG00000183617 | *MRPL54* |
| ENSG00000161013 | *MGAT4B* | ENSG00000163923 | *RPL39L* |
| ENSG00000011009 | *LYPLA2* | ENSG00000132622 | *HSPA12B* |
| ENSG00000105669 | *COPE* | ENSG00000156398 | *SFXN2* |
| ENSG00000138435 | *CHRNA1* | ENSG00000197837 | *HIST4H4* |
| ENSG00000074416 | *MGLL* | ENSG00000151136 | *BTBD11* |
| ENSG00000247596 | *TWF2* | ENSG00000151572 | *ANO4* |
| ENSG00000164796 | *CSMD3* | ENSG00000180787 | *ZFP3* |
| ENSG00000166897 | *ELFN2* | ENSG00000202198 | *RN7SK* |
| ENSG00000164484 | *TMEM200A* | ENSG00000161677 | *JOSD2* |
| ENSG00000079156 | *OSBPL6* | ENSG00000161618 | *ALDH16A1* |
| ENSG00000156804 | *FBXO32* | ENSG00000164692 | *COL1A2* |
| ENSG00000178695 | *KCTD12* | ENSG00000168542 | *COL3A1* |
| ENSG00000164849 | *GPR146* | ENSG00000187498 | *COL4A1* |
| ENSG00000157107 | *FCHO2* | ENSG00000183856 | *IQGAP3* |
| ENSG00000186446 | *ZNF501* | ENSG00000134871 | *COL4A2* |
| ENSG00000164932 | *CTHRC1* | ENSG00000130635 | *COL5A1* |
| ENSG00000166265 | *CYYR1* | ENSG00000163092 | *XIRP2* |
| ENSG00000150551 | *LYPD1* | ENSG00000144810 | *COL8A1* |
| ENSG00000135842 | *FAM129A* | ENSG00000111799 | *COL12A1* |
| ENSG00000132622 | *HSPA12B* | ENSG00000065618 | *COL17A1* |
| ENSG00000131584 | *ACAP3* | ENSG00000175182 | *FAM131A* |
| ENSG00000131386 | *GALNT15* | ENSG00000164509 | *IL31RA* |
| ENSG00000106367 | *AP1S1* | ENSG00000164318 | *EGFLAM* |
| ENSG00000178338 | *ZNF354B* | ENSG00000197261 | *C6orf141* |
| ENSG00000163297 | *ANTXR2* | ENSG00000163751 | *CPA3* |
| ENSG00000120885 | *CLU* | ENSG00000109472 | *CPE* |
| ENSG00000139263 | *LRIG3* | ENSG00000181885 | *CLDN7* |
| ENSG00000151136 | *BTBD11* | ENSG00000155975 | *VPS37A* |
| ENSG00000151572 | *ANO4* | ENSG00000165071 | *TMEM71* |
| ENSG00000172590 | *MRPL52* | ENSG00000188643 | *S100A16* |
| ENSG00000139926 | *FRMD6* | ENSG00000101331 | *CCM2L* |
| ENSG00000167703 | *SLC43A2* | ENSG00000179630 | *LACC1* |
| ENSG00000161091 | *MFSD12* | ENSG00000139971 | *C14orf37* |
| ENSG00000169991 | *IFFO2* | ENSG00000216588 | *IGSF23* |
| ENSG00000197982 | *C1orf122* | ENSG00000161888 | *SPC24* |
| ENSG00000164692 | *COL1A2* | ENSG00000118523 | *CTGF* |
| ENSG00000187498 | *COL4A1* | ENSG00000116761 | *CTH* |
| ENSG00000134871 | *COL4A2* | ENSG00000186281 | *GPAT2* |
| ENSG00000130635 | *COL5A1* | ENSG00000143869 | *GDF7* |
| ENSG00000204262 | *COL5A2* | ENSG00000091986 | *CCDC80* |
| ENSG00000163092 | *XIRP2* | ENSG00000256043 | *CTSO* |
| ENSG00000162944 | *RFTN2* | ENSG00000180611 | *MB21D2* |
| ENSG00000197467 | *COL13A1* | ENSG00000163131 | *CTSS* |
| ENSG00000057019 | *DCBLD2* | ENSG00000137075 | *RNF38* |
| ENSG00000145247 | *OCIAD2* | ENSG00000122694 | *GLIPR2* |
| ENSG00000151466 | *SCLT1* | ENSG00000174899 | *PQLC2L* |
| ENSG00000254535 | *PABPC4L* | ENSG00000154639 | *CXADR* |
| ENSG00000164284 | *GRPEL2* | ENSG00000178343 | *SHISA3* |
| ENSG00000163751 | *CPA3* | ENSG00000008283 | *CYB561* |
| ENSG00000180938 | *ZNF572* | ENSG00000051523 | *CYBA* |
| ENSG00000198832 | *SELENOM* | ENSG00000196715 | *VKORC1L1* |
| ENSG00000125995 | *ROMO1* | ENSG00000184661 | *CDCA2* |
| ENSG00000152404 | *CWF19L2* | ENSG00000183354 | *KIAA2026* |
| ENSG00000178882 | *RFLNA* | ENSG00000153071 | *DAB2* |
| ENSG00000167767 | *KRT80* | ENSG00000196352 | *CD55* |
| ENSG00000198324 | *FAM109A* | ENSG00000166750 | *SLFN5* |
| ENSG00000140511 | *HAPLN3* | ENSG00000167554 | *ZNF610* |
| ENSG00000038427 | *VCAN* | ENSG00000123977 | *DAW1* |
| ENSG00000159176 | *CSRP1* | ENSG00000173200 | *PARP15* |
| ENSG00000101439 | *CST3* | ENSG00000178175 | *ZNF366* |
| ENSG00000216588 | *IGSF23* | ENSG00000204161 | *C10orf128* |
| ENSG00000196659 | *TTC30B* | ENSG00000130511 | *SSBP4* |
| ENSG00000164733 | *CTSB* | ENSG00000140873 | *ADAMTS18* |
| ENSG00000213160 | *KLHL23* | ENSG00000228716 | *DHFR* |
| ENSG00000143869 | *GDF7* | ENSG00000147202 | *DIAPH2* |
| ENSG00000091986 | *CCDC80* | ENSG00000211448 | *DIO2* |
| ENSG00000180611 | *MB21D2* | ENSG00000075711 | *DLG1* |
| ENSG00000163131 | *CTSS* | ENSG00000198947 | *DMD* |
| ENSG00000154639 | *CXADR* | ENSG00000124721 | *DNAH8* |
| ENSG00000164463 | *CREBRF* | ENSG00000115325 | *DOK1* |
| ENSG00000051523 | *CYBA* | ENSG00000183044 | *ABAT* |
| ENSG00000153721 | *CNKSR3* | ENSG00000113070 | *HBEGF* |
| ENSG00000171115 | *GIMAP8* | ENSG00000107404 | *DVL1* |
| ENSG00000168672 | *FAM84B* | ENSG00000213694 | *S1PR3* |
| ENSG00000176853 | *FAM91A1* | ENSG00000078401 | *EDN1* |
| ENSG00000153071 | *DAB2* | ENSG00000136160 | *EDNRB* |
| ENSG00000176438 | *SYNE3* | ENSG00000247077 | *PGAM5* |
| ENSG00000176435 | *CLEC14A* | ENSG00000237624 | *OXCT2P1* |
| ENSG00000167657 | *DAPK3* | ENSG00000090776 | *EFNB1* |
| ENSG00000159640 | *ACE* | ENSG00000125266 | *EFNB2* |
| ENSG00000179144 | *GIMAP7* | ENSG00000106780 | *MEGF9* |
| ENSG00000181444 | *ZNF467* | ENSG00000165244 | *ZNF367* |
| ENSG00000213203 | *GIMAP1* | ENSG00000203805 | *PLPP4* |
| ENSG00000172893 | *DHCR7* | ENSG00000161996 | *WDR90* |
| ENSG00000211448 | *DIO2* | ENSG00000175899 | *A2M* |
| ENSG00000075711 | *DLG1* | ENSG00000163584 | *RPL22L1* |
| ENSG00000124721 | *DNAH8* | ENSG00000185909 | *KLHDC8B* |
| ENSG00000013563 | *DNASE1L1* | ENSG00000159314 | *ARHGAP27* |
| ENSG00000197102 | *DYNC1H1* | ENSG00000169689 | *CENPX* |
| ENSG00000119772 | *DNMT3A* | ENSG00000169683 | *LRRC45* |
| ENSG00000197635 | *DPP4* | ENSG00000074800 | *ENO1* |
| ENSG00000157514 | *TSC22D3* | ENSG00000123572 | *NRK* |
| ENSG00000134769 | *DTNA* | ENSG00000145242 | *EPHA5* |
| ENSG00000113070 | *HBEGF* | ENSG00000225383 | *SFTA1P* |
| ENSG00000120129 | *DUSP1* | ENSG00000157554 | *ERG* |
| ENSG00000133740 | *E2F5* | ENSG00000148218 | *ALAD* |
| ENSG00000078401 | *EDN1* | ENSG00000173153 | *ESRRA* |
| ENSG00000111752 | *PHC1* | ENSG00000182197 | *EXT1* |
| ENSG00000125266 | *EFNB2* | ENSG00000165092 | *ALDH1A1* |
| ENSG00000106546 | *AHR* | ENSG00000144554 | *FANCD2* |
| ENSG00000111145 | *ELK3* | ENSG00000078098 | *FAP* |
| ENSG00000158711 | *ELK4* | ENSG00000177409 | *SAMD9L* |
| ENSG00000169621 | *APLF* | ENSG00000083857 | *FAT1* |
| ENSG00000154240 | *CEP112* | ENSG00000107731 | *UNC5B* |
| ENSG00000134531 | *EMP1* | ENSG00000184254 | *ALDH1A3* |
| ENSG00000213853 | *EMP2* | ENSG00000184232 | *OAF* |
| ENSG00000142227 | *EMP3* | ENSG00000172738 | *TMEM217* |
| ENSG00000170006 | *TMEM154* | ENSG00000160813 | *PPP1R35* |
| ENSG00000112297 | *AIM1* | ENSG00000106009 | *BRAT1* |
| ENSG00000106991 | *ENG* | ENSG00000146555 | *SDK1* |
| ENSG00000183323 | *CCDC125* | ENSG00000189134 | *NKAPL* |
| ENSG00000116016 | *EPAS1* | ENSG00000166924 | *NYAP1* |
| ENSG00000145242 | *EPHA5* | ENSG00000161513 | *FDXR* |
| ENSG00000151491 | *EPS8* | ENSG00000168496 | *FEN1* |
| ENSG00000213462 | *ERV3-1* | ENSG00000138675 | *FGF5* |
| ENSG00000173153 | *ESRRA* | ENSG00000179399 | *GPC5* |
| ENSG00000182197 | *EXT1* | ENSG00000087303 | *NID2* |
| ENSG00000164251 | *F2RL1* | ENSG00000158186 | *MRAS* |
| ENSG00000068366 | *ACSL4* | ENSG00000162631 | *NTNG1* |
| ENSG00000078098 | *FAP* | ENSG00000001561 | *ENPP4* |
| ENSG00000169710 | *FASN* | ENSG00000135299 | *ANKRD6* |
| ENSG00000083857 | *FAT1* | ENSG00000141337 | *ARSG* |
| ENSG00000183621 | *ZNF438* | ENSG00000118985 | *ELL2* |
| ENSG00000069122 | *ADGRF5* | ENSG00000129116 | *PALLD* |
| ENSG00000172738 | *TMEM217* | ENSG00000020577 | *SAMD4A* |
| ENSG00000180537 | *RNF182* | ENSG00000187239 | *FNBP1* |
| ENSG00000164651 | *SP8* | ENSG00000102935 | *ZNF423* |
| ENSG00000146555 | *SDK1* | ENSG00000123200 | *ZC3H13* |
| ENSG00000005108 | *THSD7A* | ENSG00000082397 | *EPB41L3* |
| ENSG00000180354 | *MTURN* | ENSG00000100304 | *TTLL12* |
| ENSG00000161040 | *FBXL13* | ENSG00000107104 | *KANK1* |
| ENSG00000153993 | *SEMA3D* | ENSG00000137573 | *SULF1* |
| ENSG00000138685 | *FGF2* | ENSG00000160796 | *NBEAL2* |
| ENSG00000138675 | *FGF5* | ENSG00000171735 | *CAMTA1* |
| ENSG00000149925 | *ALDOA* | ENSG00000049759 | *NEDD4L* |
| ENSG00000105967 | *TFEC* | ENSG00000131018 | *SYNE1* |
| ENSG00000135315 | *CEP162* | ENSG00000115414 | *FN1* |
| ENSG00000071246 | *VASH1* | ENSG00000121152 | *NCAPH* |
| ENSG00000107890 | *ANKRD26* | ENSG00000169946 | *ZFPM2* |
| ENSG00000169760 | *NLGN1* | ENSG00000127603 | *MACF1* |
| ENSG00000096060 | *FKBP5* | ENSG00000179604 | *CDC42EP4* |
| ENSG00000064932 | *SBNO2* | ENSG00000011454 | *RABGAP1* |
| ENSG00000138722 | *MMRN1* | ENSG00000133265 | *HSPBP1* |
| ENSG00000118985 | *ELL2* | ENSG00000130147 | *SH3BP4* |
| ENSG00000107984 | *DKK1* | ENSG00000123892 | *RAB38* |
| ENSG00000054598 | *FOXC1* | ENSG00000128228 | *SDF2L1* |
| ENSG00000085831 | *TTC39A* | ENSG00000125848 | *FLRT3* |
| ENSG00000020577 | *SAMD4A* | ENSG00000198691 | *ABCA4* |
| ENSG00000187239 | *FNBP1* | ENSG00000102384 | *CENPI* |
| ENSG00000150907 | *FOXO1* | ENSG00000180071 | *ANKRD18A* |
| ENSG00000102935 | *ZNF423* | ENSG00000126709 | *IFI6* |
| ENSG00000151702 | *FLI1* | ENSG00000143469 | *SYT14* |
| ENSG00000109436 | *TBC1D9* | ENSG00000186994 | *KANK3* |
| ENSG00000010327 | *STAB1* | ENSG00000141424 | *SLC39A6* |
| ENSG00000011523 | *CEP68* | ENSG00000130520 | *LSM4* |
| ENSG00000019144 | *PHLDB1* | ENSG00000117308 | *GALE* |
| ENSG00000137573 | *SULF1* | ENSG00000108479 | *GALK1* |
| ENSG00000054654 | *SYNE2* | ENSG00000185432 | *METTL7A* |
| ENSG00000037280 | *FLT4* | ENSG00000205356 | *TECPR1* |
| ENSG00000186866 | *POFUT2* | ENSG00000164087 | *POC1A* |
| ENSG00000064999 | *ANKS1A* | ENSG00000196155 | *PLEKHG4* |
| ENSG00000131018 | *SYNE1* | ENSG00000197555 | *SIPA1L1* |
| ENSG00000103187 | *COTL1* | ENSG00000152217 | *SETBP1* |
| ENSG00000107130 | *NCS1* | ENSG00000198624 | *CCDC69* |
| ENSG00000154262 | *ABCA6* | ENSG00000181722 | *ZBTB20* |
| ENSG00000154589 | *LY96* | ENSG00000119403 | *PHF19* |
| ENSG00000103066 | *PLA2G15* | ENSG00000137507 | *LRRC32* |
| ENSG00000117758 | *STX12* | ENSG00000161956 | *SENP3* |
| ENSG00000185022 | *MAFF* | ENSG00000154027 | *AK5* |
| ENSG00000125848 | *FLRT3* | ENSG00000131979 | *GCH1* |
| ENSG00000099968 | *BCL2L13* | ENSG00000135441 | *BLOC1S1* |
| ENSG00000137409 | *MTCH1* | ENSG00000100558 | *PLEK2* |
| ENSG00000110218 | *PANX1* | ENSG00000099800 | *TIMM13* |
| ENSG00000172031 | *EPHX4* | ENSG00000134809 | *TIMM10* |
| ENSG00000126709 | *IFI6* | ENSG00000238578 | *SNORD4A* |
| ENSG00000184465 | *WDR27* | ENSG00000225091 | *SNORA71A* |
| ENSG00000137700 | *SLC37A4* | ENSG00000204136 | *GGTA1P* |
| ENSG00000166046 | *TCP11L2* | ENSG00000200959 | *SNORA74A* |
| ENSG00000143469 | *SYT14* | ENSG00000200156 | *RNU5B-1* |
| ENSG00000184005 | *ST6GALNAC3* | ENSG00000070756 | *PABPC1* |
| ENSG00000165072 | *MAMDC2* | ENSG00000143322 | *ABL2* |
| ENSG00000172159 | *FRMD3* | ENSG00000265107 | *GJA5* |
| ENSG00000172260 | *NEGR1* | ENSG00000148677 | *ANKRD1* |
| ENSG00000151014 | *NOCT* | ENSG00000105327 | *BBC3* |
| ENSG00000198792 | *TMEM184B* | ENSG00000050165 | *DKK3* |
| ENSG00000185432 | *METTL7A* | ENSG00000107719 | *PALD1* |
| ENSG00000196155 | *PLEKHG4* | ENSG00000118407 | *FILIP1* |
| ENSG00000143641 | *GALNT2* | ENSG00000250510 | *GPR162* |
| ENSG00000170011 | *MYRIP* | ENSG00000146072 | *TNFRSF21* |
| ENSG00000198795 | *ZNF521* | ENSG00000150593 | *PDCD4* |
| ENSG00000066279 | *ASPM* | ENSG00000118946 | *PCDH17* |
| ENSG00000104951 | *IL4I1* | ENSG00000102359 | *SRPX2* |
| ENSG00000132424 | *PNISR* | ENSG00000154553 | *PDLIM3* |
| ENSG00000020181 | *ADGRA2* | ENSG00000078053 | *AMPH* |
| ENSG00000197324 | *LRP10* | ENSG00000178445 | *GLDC* |
| ENSG00000138604 | *GLCE* | ENSG00000137502 | *RAB30* |
| ENSG00000152217 | *SETBP1* | ENSG00000108106 | *UBE2S* |
| ENSG00000109046 | *WSB1* | ENSG00000244509 | *APOBEC3C* |
| ENSG00000137507 | *LRRC32* | ENSG00000136717 | *BIN1* |
| ENSG00000066735 | *KIF26A* | ENSG00000211450 | *SELENOH* |
| ENSG00000157214 | *STEAP2* | ENSG00000250303 | *LOC283140* |
| ENSG00000134242 | *PTPN22* | ENSG00000149380 | *P4HA3* |
| ENSG00000154217 | *PITPNC1* | ENSG00000165434 | *PGM2L1* |
| ENSG00000092621 | *PHGDH* | ENSG00000177990 | *DPY19L2* |
| ENSG00000146021 | *KLHL3* | ENSG00000255874 | *LINC00346* |
| ENSG00000154027 | *AK5* | ENSG00000139508 | *SLC46A3* |
| ENSG00000177628 | *GBA* | ENSG00000154188 | *ANGPT1* |
| ENSG00000114480 | *GBE1* | ENSG00000154874 | *CCDC144B* |
| ENSG00000152137 | *HSPB8* | ENSG00000224877 | *NDUFAF8* |
| ENSG00000106852 | *LHX6* | ENSG00000227195 | *MIR663AHG* |
| ENSG00000187210 | *GCNT1* | ENSG00000091879 | *ANGPT2* |
| ENSG00000172927 | *MYEOV* | ENSG00000144792 | *ZNF660* |
| ENSG00000185352 | *HS6ST3* | ENSG00000099875 | *MKNK2* |
| ENSG00000204136 | *GGTA1P* | ENSG00000233276 | *GPX1* |
| ENSG00000200959 | *SNORA74A* | ENSG00000115290 | *GRB14* |
| ENSG00000099998 | *GGT5* | ENSG00000102760 | *RGCC* |
| ENSG00000164647 | *STEAP1* | ENSG00000168701 | *TMEM208* |
| ENSG00000196511 | *TPK1* | ENSG00000120217 | *CD274* |
| ENSG00000265107 | *GJA5* | ENSG00000161800 | *RACGAP1* |
| ENSG00000148677 | *ANKRD1* | ENSG00000276043 | *UHRF1* |
| ENSG00000078081 | *LAMP3* | ENSG00000163739 | *CXCL1* |
| ENSG00000114861 | *FOXP1* | ENSG00000025770 | *NCAPH2* |
| ENSG00000050165 | *DKK3* | ENSG00000164116 | *GUCY1A3* |
| ENSG00000102393 | *GLA* | ENSG00000061918 | *GUCY1B3* |
| ENSG00000198075 | *SULT1C4* | ENSG00000121957 | *GPSM2* |
| ENSG00000102359 | *SRPX2* | ENSG00000160447 | *PKN3* |
| ENSG00000130768 | *SMPDL3B* | ENSG00000136732 | *GYPC* |
| ENSG00000108106 | *UBE2S* | ENSG00000197019 | *SERTAD1* |
| ENSG00000250303 | *LOC283140* | ENSG00000089486 | *CDIP1* |
| ENSG00000171435 | *KSR2* | ENSG00000123636 | *BAZ2B* |
| ENSG00000139508 | *SLC46A3* | ENSG00000147010 | *SH3KBP1* |
| ENSG00000099814 | *CEP170B* | ENSG00000187837 | *HIST1H1C* |
| ENSG00000166450 | *PRTG* | ENSG00000124575 | *HIST1H1D* |
| ENSG00000180229 | *HERC2P3* | ENSG00000184357 | *HIST1H1B* |
| ENSG00000154188 | *ANGPT1* | ENSG00000277075 | *HIST1H2AE* |
| ENSG00000186665 | *C17orf58* | ENSG00000188486 | *H2AFX* |
| ENSG00000154874 | *CCDC144B* | ENSG00000164032 | *H2AFZ* |
| ENSG00000196268 | *ZNF493* | ENSG00000158373 | *HIST1H2BD* |
| ENSG00000267519 | *LOC284454* | ENSG00000276410 | *HIST1H2BB* |
| ENSG00000227195 | *MIR663AHG* | ENSG00000124610 | *HIST1H1A* |
| ENSG00000198719 | *DLL1* | ENSG00000099937 | *SERPIND1* |
| ENSG00000180376 | *CCDC66* | ENSG00000000971 | *CFH* |
| ENSG00000249464 | *LINC01091* | ENSG00000013016 | *EHD3* |
| ENSG00000126882 | *FAM78A* | ENSG00000177374 | *HIC1* |
| ENSG00000233276 | *GPX1* | ENSG00000095951 | *HIVEP1* |
| ENSG00000081087 | *OSTM1* | ENSG00000231389 | *HLA-DPA1* |
| ENSG00000087884 | *AAMDC* | ENSG00000223865 | *HLA-DPB1* |
| ENSG00000102760 | *RGCC* | ENSG00000138356 | *AOX1* |
| ENSG00000163884 | *KLF15* | ENSG00000072571 | *HMMR* |
| ENSG00000166825 | *ANPEP* | ENSG00000100292 | *HMOX1* |
| ENSG00000120217 | *CD274* | ENSG00000129514 | *FOXA1* |
| ENSG00000163739 | *CXCL1* | ENSG00000184270 | *HIST2H2AB* |
| ENSG00000213366 | *GSTM2* | ENSG00000107282 | *APBA1* |
| ENSG00000100027 | *YPEL1* | ENSG00000180806 | *HOXC9* |
| ENSG00000164116 | *GUCY1A3* | ENSG00000126457 | *PRMT1* |
| ENSG00000061918 | *GUCY1B3* | ENSG00000086696 | *HSD17B2* |
| ENSG00000106266 | *SNX8* | ENSG00000089685 | *BIRC5* |
| ENSG00000197019 | *SERTAD1* | ENSG00000135914 | *HTR2B* |
| ENSG00000124593 | *PRICKLE4* | ENSG00000090339 | *ICAM1* |
| ENSG00000089486 | *CDIP1* | ENSG00000108622 | *ICAM2* |
| ENSG00000147650 | *LRP12* | ENSG00000254004 | *ZNF260* |
| ENSG00000151967 | *SCHIP1* | ENSG00000100036 | *SLC35E4* |
| ENSG00000189060 | *H1F0* | ENSG00000125968 | *ID1* |
| ENSG00000187837 | *HIST1H1C* | ENSG00000010404 | *IDS* |
| ENSG00000168298 | *HIST1H1E* | ENSG00000205403 | *CFI* |
| ENSG00000182718 | *ANXA2* | ENSG00000147036 | *LANCL3* |
| ENSG00000063854 | *HAGH* | ENSG00000130203 | *APOE* |
| ENSG00000099937 | *SERPIND1* | ENSG00000140443 | *IGF1R* |
| ENSG00000138772 | *ANXA3* | ENSG00000115457 | *IGFBP2* |
| ENSG00000000971 | *CFH* | ENSG00000167779 | *IGFBP6* |
| ENSG00000213977 | *TAX1BP3* | ENSG00000163453 | *IGFBP7* |
| ENSG00000127946 | *HIP1* | ENSG00000198931 | *APRT* |
| ENSG00000164104 | *HMGB2* | ENSG00000115008 | *IL1A* |
| ENSG00000138356 | *AOX1* | ENSG00000115594 | *IL1R1* |
| ENSG00000103415 | *HMOX2* | ENSG00000131724 | *IL13RA1* |
| ENSG00000163412 | *EIF4E3* | ENSG00000122641 | *INHBA* |
| ENSG00000180806 | *HOXC9* | ENSG00000163083 | *INHBB* |
| ENSG00000115756 | *HPCAL1* | ENSG00000117595 | *IRF6* |
| ENSG00000086696 | *HSD17B2* | ENSG00000115232 | *ITGA4* |
| ENSG00000090339 | *ICAM1* | ENSG00000138448 | *ITGAV* |
| ENSG00000148488 | *ST8SIA6* | ENSG00000259207 | *ITGB3* |
| ENSG00000197841 | *ZNF181* | ENSG00000132470 | *ITGB4* |
| ENSG00000185860 | *CCDC190* | ENSG00000105855 | *ITGB8* |
| ENSG00000165949 | *IFI27* | ENSG00000182118 | *FAM89A* |
| ENSG00000122483 | *CCDC18* | ENSG00000135976 | *ANKRD36* |
| ENSG00000162687 | *KCNT2* | ENSG00000069020 | *MAST4* |
| ENSG00000176771 | *NCKAP5* | ENSG00000157404 | *KIT* |
| ENSG00000234284 | *ZNF879* | ENSG00000237649 | *KIFC1* |
| ENSG00000188107 | *EYS* | ENSG00000079616 | *KIF22* |
| ENSG00000157593 | *SLC35B2* | ENSG00000186081 | *KRT5* |
| ENSG00000168528 | *SERINC2* | ENSG00000205420 | *KRT6A* |
| ENSG00000130203 | *APOE* | ENSG00000171401 | *KRT13* |
| ENSG00000242498 | *ARPIN* | ENSG00000203760 | *CENPW* |
| ENSG00000115457 | *IGFBP2* | ENSG00000171345 | *KRT19* |
| ENSG00000167779 | *IGFBP6* | ENSG00000175155 | *YPEL2* |
| ENSG00000163453 | *IGFBP7* | ENSG00000264343 | *NOTCH2NL* |
| ENSG00000142871 | *CYR61* | ENSG00000168887 | *C2orf68* |
| ENSG00000198931 | *APRT* | ENSG00000206538 | *VGLL3* |
| ENSG00000115008 | *IL1A* | ENSG00000115963 | *RND3* |
| ENSG00000115594 | *IL1R1* | ENSG00000130702 | *LAMA5* |
| ENSG00000185291 | *IL3RA* | ENSG00000091136 | *LAMB1* |
| ENSG00000136244 | *IL6* | ENSG00000058085 | *LAMC2* |
| ENSG00000168685 | *IL7R* | ENSG00000156466 | *GDF6* |
| ENSG00000169429 | *CXCL8* | ENSG00000182541 | *LIMK2* |
| ENSG00000240583 | *AQP1* | ENSG00000169756 | *LIMS1* |
| ENSG00000163083 | *INHBB* | ENSG00000079435 | *LIPE* |
| ENSG00000171105 | *INSR* | ENSG00000160789 | *LMNA* |
| ENSG00000169083 | *AR* | ENSG00000113368 | *LMNB1* |
| ENSG00000005884 | *ITGA3* | ENSG00000197182 | *MIRLET7BHG* |
| ENSG00000115232 | *ITGA4* | ENSG00000186479 | *RGS7BP* |
| ENSG00000161638 | *ITGA5* | ENSG00000177570 | *SAMD12* |
| ENSG00000138448 | *ITGAV* | ENSG00000188921 | *HACD4* |
| ENSG00000259207 | *ITGB3* | ENSG00000182489 | *XKRX* |
| ENSG00000132470 | *ITGB4* | ENSG00000119681 | *LTBP2* |
| ENSG00000242372 | *EIF6* | ENSG00000164109 | *MAD2L1* |
| ENSG00000082781 | *ITGB5* | ENSG00000101846 | *STS* |
| ENSG00000105855 | *ITGB8* | ENSG00000111885 | *MAN1A1* |
| ENSG00000184916 | *JAG2* | ENSG00000132561 | *MATN2* |
| ENSG00000162434 | *JAK1* | ENSG00000103495 | *MAZ* |
| ENSG00000177606 | *JUN* | ENSG00000073111 | *MCM2* |
| ENSG00000182118 | *FAM89A* | ENSG00000104738 | *MCM4* |
| ENSG00000135976 | *ANKRD36* | ENSG00000100297 | *MCM5* |
| ENSG00000188157 | *AGRN* | ENSG00000076003 | *MCM6* |
| ENSG00000143603 | *KCNN3* | ENSG00000166508 | *MCM7* |
| ENSG00000140859 | *KIFC3* | ENSG00000111339 | *ART4* |
| ENSG00000186081 | *KRT5* | ENSG00000095015 | *MAP3K1* |
| ENSG00000135480 | *KRT7* | ENSG00000049130 | *KITLG* |
| ENSG00000171401 | *KRT13* | ENSG00000170430 | *MGMT* |
| ENSG00000171346 | *KRT15* | ENSG00000002586 | *CD99* |
| ENSG00000181350 | *LRRC75A* | ENSG00000187098 | *MITF* |
| ENSG00000253368 | *TRNP1* | ENSG00000087245 | *MMP2* |
| ENSG00000155366 | *RHOC* | ENSG00000166670 | *MMP10* |
| ENSG00000206538 | *VGLL3* | ENSG00000204899 | *MZT1* |
| ENSG00000115963 | *RND3* | ENSG00000206149 | *HERC2P9* |
| ENSG00000185896 | *LAMP1* | ENSG00000203995 | *ZYG11A* |
| ENSG00000058085 | *LAMC2* | ENSG00000215146 | *LOC441666* |
| ENSG00000156466 | *GDF6* | ENSG00000198899 | *ATP6* |
| ENSG00000130164 | *LDLR* | ENSG00000228253 | *ATP8* |
| ENSG00000116678 | *LEPR* | ENSG00000198804 | *COX1* |
| ENSG00000100097 | *LGALS1* | ENSG00000198712 | *COX2* |
| ENSG00000113594 | *LIFR* | ENSG00000198938 | *COX3* |
| ENSG00000099204 | *ABLIM1* | ENSG00000198727 | *CYTB* |
| ENSG00000221968 | *FADS3* | ENSG00000100714 | *MTHFD1* |
| ENSG00000135363 | *LMO2* | ENSG00000198888 | *ND1* |
| ENSG00000136153 | *LMO7* | ENSG00000198763 | *ND2* |
| ENSG00000124374 | *PAIP2B* | ENSG00000198840 | *ND3* |
| ENSG00000184305 | *CCSER1* | ENSG00000198886 | *ND4* |
| ENSG00000186479 | *RGS7BP* | ENSG00000212907 | *ND4L* |
| ENSG00000134013 | *LOXL2* | ENSG00000198786 | *ND5* |
| ENSG00000163956 | *LRPAP1* | ENSG00000157601 | *MX1* |
| ENSG00000154102 | *C16orf74* | ENSG00000099860 | *GADD45B* |
| ENSG00000160285 | *LSS* | ENSG00000133026 | *MYH10* |
| ENSG00000111144 | *LTA4H* | ENSG00000176658 | *MYO1D* |
| ENSG00000119681 | *LTBP2* | ENSG00000157483 | *MYO1E* |
| ENSG00000226380 | *MIR29A* | ENSG00000196586 | *MYO6* |
| ENSG00000120693 | *SMAD9* | ENSG00000137474 | *MYO7A* |
| ENSG00000100299 | *ARSA* | ENSG00000145555 | *MYO10* |
| ENSG00000166963 | *MAP1A* | ENSG00000117650 | *NEK2* |
| ENSG00000132561 | *MATN2* | ENSG00000103024 | *NME3* |
| ENSG00000076706 | *MCAM* | ENSG00000164867 | *NOS3* |
| ENSG00000106511 | *MEOX2* | ENSG00000169418 | *NPR1* |
| ENSG00000049130 | *KITLG* | ENSG00000065057 | *NTHL1* |
| ENSG00000111341 | *MGP* | ENSG00000185483 | *ROR1* |
| ENSG00000143198 | *MGST3* | ENSG00000115758 | *ODC1* |
| ENSG00000104763 | *ASAH1* | ENSG00000116213 | *WRAP73* |
| ENSG00000204516 | *MICB* | ENSG00000085840 | *ORC1* |
| ENSG00000171843 | *MLLT3* | ENSG00000086991 | *NOX4* |
| ENSG00000196611 | *MMP1* | ENSG00000106366 | *SERPINE1* |
| ENSG00000166670 | *MMP10* | ENSG00000197632 | *SERPINB2* |
| ENSG00000157227 | *MMP14* | ENSG00000149269 | *PAK1* |
| ENSG00000119711 | *ALDH6A1* | ENSG00000204387 | *C6orf48* |
| ENSG00000182534 | *MXRA7* | ENSG00000173599 | *PC* |
| ENSG00000015133 | *CCDC88C* | ENSG00000169851 | *PCDH7* |
| ENSG00000206149 | *HERC2P9* | ENSG00000184226 | *PCDH9* |
| ENSG00000169715 | *MT1E* | ENSG00000078674 | *PCM1* |
| ENSG00000260549 | *MT1L* | ENSG00000132646 | *PCNA* |
| ENSG00000125148 | *MT2A* | ENSG00000053372 | *MRTO4* |
| ENSG00000167508 | *MVD* | ENSG00000172889 | *EGFL7* |
| ENSG00000157601 | *MX1* | ENSG00000109576 | *AADAT* |
| ENSG00000133026 | *MYH10* | ENSG00000136463 | *TACO1* |
| ENSG00000092841 | *MYL6* | ENSG00000108798 | *ABI3* |
| ENSG00000183091 | *NEB* | ENSG00000099139 | *PCSK5* |
| ENSG00000090266 | *NDUFB2* | ENSG00000150048 | *CLEC1A* |
| ENSG00000129559 | *NEDD8* | ENSG00000171222 | *SCAND1* |
| ENSG00000111859 | *NEDD9* | ENSG00000167775 | *CD320* |
| ENSG00000136098 | *NEK3* | ENSG00000113555 | *PCDH12* |
| ENSG00000186575 | *NF2* | ENSG00000124785 | *NRN1* |
| ENSG00000143153 | *ATP1B1* | ENSG00000115252 | *PDE1A* |
| ENSG00000116962 | *NID1* | ENSG00000154678 | *PDE1C* |
| ENSG00000198805 | *PNP* | ENSG00000186642 | *PDE2A* |
| ENSG00000144061 | *NPHP1* | ENSG00000187688 | *TRPV2* |
| ENSG00000169418 | *NPR1* | ENSG00000184588 | *PDE4B* |
| ENSG00000185483 | *ROR1* | ENSG00000075218 | *GTSE1* |
| ENSG00000111335 | *OAS2* | ENSG00000171425 | *ZNF581* |
| ENSG00000013297 | *CLDN11* | ENSG00000100311 | *PDGFB* |
| ENSG00000135124 | *P2RX4* | ENSG00000129048 | *ACKR4* |
| ENSG00000140564 | *FURIN* | ENSG00000013306 | *SLC25A39* |
| ENSG00000117450 | *PRDX1* | ENSG00000119801 | *YPEL5* |
| ENSG00000106366 | *SERPINE1* | ENSG00000131153 | *GINS2* |
| ENSG00000197632 | *SERPINB2* | ENSG00000197594 | *ENPP1* |
| ENSG00000185630 | *PBX1* | ENSG00000164035 | *EMCN* |
| ENSG00000142546 | *NOSIP* | ENSG00000161980 | *POLR3K* |
| ENSG00000109618 | *SEPSECS* | ENSG00000100625 | *SIX4* |
| ENSG00000167772 | *ANGPTL4* | ENSG00000178921 | *PFAS* |
| ENSG00000189159 | *HN1* | ENSG00000135919 | *SERPINE2* |
| ENSG00000023902 | *PLEKHO1* | ENSG00000105851 | *PIK3CG* |
| ENSG00000137804 | *NUSAP1* | ENSG00000008710 | *PKD1* |
| ENSG00000175426 | *PCSK1* | ENSG00000057294 | *PKP2* |
| ENSG00000154781 | *CCDC174* | ENSG00000117410 | *ATP6V0B* |
| ENSG00000006327 | *TNFRSF12A* | ENSG00000136404 | *TM6SF1* |
| ENSG00000137877 | *SPTBN5* | ENSG00000166851 | *PLK1* |
| ENSG00000115252 | *PDE1A* | ENSG00000071655 | *MBD3* |
| ENSG00000182022 | *CHST15* | ENSG00000165240 | *ATP7A* |
| ENSG00000154678 | *PDE1C* | ENSG00000102575 | *ACP5* |
| ENSG00000100554 | *ATP6V1D* | ENSG00000184702 | *SEPT5* |
| ENSG00000172572 | *PDE3A* | ENSG00000172965 | *MIR4435-2HG* |
| ENSG00000187688 | *TRPV2* | ENSG00000128567 | *PODXL* |
| ENSG00000196247 | *ZNF107* | ENSG00000062822 | *POLD1* |
| ENSG00000168077 | *SCARA3* | ENSG00000168002 | *POLR2G* |
| ENSG00000143476 | *DTL* | ENSG00000170891 | *CYTL1* |
| ENSG00000100311 | *PDGFB* | ENSG00000129195 | *FAM64A* |
| ENSG00000129048 | *ACKR4* | ENSG00000145390 | *USP53* |
| ENSG00000117543 | *DPH5* | ENSG00000168209 | *DDIT4* |
| ENSG00000004799 | *PDK4* | ENSG00000085224 | *ATRX* |
| ENSG00000171314 | *PGAM1* | ENSG00000099364 | *FBXL19* |
| ENSG00000119630 | *PGF* | ENSG00000196544 | *BORCS6* |
| ENSG00000135919 | *SERPINE2* | ENSG00000186871 | *ERCC6L* |
| ENSG00000133056 | *PIK3C2B* | ENSG00000059769 | *DNAJC25* |
| ENSG00000171608 | *PIK3CD* | ENSG00000099260 | *PALMD* |
| ENSG00000067225 | *PKM* | ENSG00000146918 | *NCAPG2* |
| ENSG00000104368 | *PLAT* | ENSG00000166912 | *MTMR10* |
| ENSG00000122861 | *PLAU* | ENSG00000105538 | *RASIP1* |
| ENSG00000011422 | *PLAUR* | ENSG00000101695 | *RNF125* |
| ENSG00000117410 | *ATP6V0B* | ENSG00000177692 | *DNAJC28* |
| ENSG00000136404 | *TM6SF1* | ENSG00000087253 | *LPCAT2* |
| ENSG00000083444 | *PLOD1* | ENSG00000130748 | *TMEM160* |
| ENSG00000152952 | *PLOD2* | ENSG00000101220 | *C20orf27* |
| ENSG00000114554 | *PLXNA1* | ENSG00000143224 | *PPOX* |
| ENSG00000071655 | *MBD3* | ENSG00000175756 | *AURKAIP1* |
| ENSG00000008277 | *ADAM22* | ENSG00000185480 | *PARPBP* |
| ENSG00000141682 | *PMAIP1* | ENSG00000170779 | *CDCA4* |
| ENSG00000102575 | *ACP5* | ENSG00000137831 | *UACA* |
| ENSG00000184702 | *SEPT5* | ENSG00000126870 | *WDR60* |
| ENSG00000108387 | *SEPT4* | ENSG00000072041 | *SLC6A15* |
| ENSG00000147481 | *SNTG1* | ENSG00000134690 | *CDCA8* |
| ENSG00000099817 | *POLR2E* | ENSG00000074964 | *ARHGEF10L* |
| ENSG00000170891 | *CYTL1* | ENSG00000138180 | *CEP55* |
| ENSG00000177700 | *POLR2L* | ENSG00000196368 | *NUDT11* |
| ENSG00000145990 | *GFOD1* | ENSG00000090530 | *P3H2* |
| ENSG00000052126 | *PLEKHA5* | ENSG00000073711 | *PPP2R3A* |
| ENSG00000033100 | *CHPF2* | ENSG00000146859 | *TMEM140* |
| ENSG00000189184 | *PCDH18* | ENSG00000038210 | *PI4K2B* |
| ENSG00000091127 | *PUS7* | ENSG00000103381 | *CPPED1* |
| ENSG00000154133 | *ROBO4* | ENSG00000104341 | *LAPTM4B* |
| ENSG00000168209 | *DDIT4* | ENSG00000123485 | *HJURP* |
| ENSG00000128917 | *DLL4* | ENSG00000095383 | *TBC1D2* |
| ENSG00000069812 | *HES2* | ENSG00000065328 | *MCM10* |
| ENSG00000005469 | *CROT* | ENSG00000225470 | *JPX* |
| ENSG00000164951 | *PDP1* | ENSG00000162545 | *CAMK2N1* |
| ENSG00000184840 | *TMED9* | ENSG00000169826 | *CSGALNACT2* |
| ENSG00000178075 | *GRAMD1C* | ENSG00000101311 | *FERMT1* |
| ENSG00000011258 | *MBTD1* | ENSG00000024526 | *DEPDC1* |
| ENSG00000137960 | *GIPC2* | ENSG00000147642 | *SYBU* |
| ENSG00000137501 | *SYTL2* | ENSG00000022556 | *NLRP2* |
| ENSG00000198113 | *TOR4A* | ENSG00000165801 | *ARHGEF40* |
| ENSG00000044459 | *CNTLN* | ENSG00000058804 | *NDC1* |
| ENSG00000118898 | *PPL* | ENSG00000198185 | *ZNF334* |
| ENSG00000101695 | *RNF125* | ENSG00000218336 | *TENM3* |
| ENSG00000175756 | *AURKAIP1* | ENSG00000105011 | *ASF1B* |
| ENSG00000141219 | *C17orf80* | ENSG00000042088 | *TDP1* |
| ENSG00000160058 | *BSDC1* | ENSG00000140563 | *MCTP2* |
| ENSG00000072041 | *SLC6A15* | ENSG00000035499 | *DEPDC1B* |
| ENSG00000134186 | *PRPF38B* | ENSG00000112304 | *ACOT13* |
| ENSG00000090530 | *P3H2* | ENSG00000168078 | *PBK* |
| ENSG00000172731 | *LRRC20* | ENSG00000197852 | *FAM212B* |
| ENSG00000137269 | *LRRC1* | ENSG00000156711 | *MAPK13* |
| ENSG00000115107 | *STEAP3* | ENSG00000145431 | *PDGFC* |
| ENSG00000147419 | *CCDC25* | ENSG00000183943 | *PRKX* |
| ENSG00000066027 | *PPP2R5A* | ENSG00000112852 | *PCDHB2* |
| ENSG00000137522 | *RNF121* | ENSG00000147697 | *GSDMC* |
| ENSG00000133574 | *GIMAP4* | ENSG00000120549 | *KIAA1217* |
| ENSG00000149923 | *PPP4C* | ENSG00000082497 | *SERTAD4* |
| ENSG00000135002 | *RFK* | ENSG00000130962 | *PRRG1* |
| ENSG00000129534 | *MIS18BP1* | ENSG00000010438 | *PRSS3* |
| ENSG00000138587 | *MNS1* | ENSG00000202515 | *VTRNA1-3* |
| ENSG00000152953 | *STK32B* | ENSG00000182916 | *TCEAL7* |
| ENSG00000104341 | *LAPTM4B* | ENSG00000176907 | *C8orf4* |
| ENSG00000163046 | *ANKRD30BL* | ENSG00000141994 | *DUS3L* |
| ENSG00000162545 | *CAMK2N1* | ENSG00000124225 | *PMEPA1* |
| ENSG00000122862 | *SRGN* | ENSG00000240065 | *PSMB9* |
| ENSG00000110675 | *ELMOD1* | ENSG00000205220 | *PSMB10* |
| ENSG00000130827 | *PLXNA3* | ENSG00000163808 | *KIF15* |
| ENSG00000135905 | *DOCK10* | ENSG00000163050 | *COQ8A* |
| ENSG00000162409 | *PRKAA2* | ENSG00000163638 | *ADAMTS9* |
| ENSG00000118242 | *MREG* | ENSG00000114698 | *PLSCR4* |
| ENSG00000116871 | *MAP7D1* | ENSG00000090971 | *NAT14* |
| ENSG00000218336 | *TENM3* | ENSG00000117643 | *MAN1C1* |
| ENSG00000167601 | *AXL* | ENSG00000134247 | *PTGFRN* |
| ENSG00000171132 | *PRKCE* | ENSG00000124212 | *PTGIS* |
| ENSG00000042317 | *SPATA7* | ENSG00000152253 | *SPC25* |
| ENSG00000065675 | *PRKCQ* | ENSG00000095303 | *PTGS1* |
| ENSG00000176871 | *WSB2* | ENSG00000073756 | *PTGS2* |
| ENSG00000186918 | *ZNF395* | ENSG00000128578 | *STRIP2* |
| ENSG00000005483 | *KMT2E* | ENSG00000186260 | *MKL2* |
| ENSG00000128833 | *MYO5C* | ENSG00000196935 | *SRGAP1* |
| ENSG00000196562 | *SULF2* | ENSG00000183775 | *KCTD16* |
| ENSG00000145431 | *PDGFC* | ENSG00000136383 | *ALPK3* |
| ENSG00000147697 | *GSDMC* | ENSG00000138650 | *PCDH10* |
| ENSG00000171867 | *PRNP* | ENSG00000138771 | *SHROOM3* |
| ENSG00000120549 | *KIAA1217* | ENSG00000013293 | *SLC7A14* |
| ENSG00000082497 | *SERTAD4* | ENSG00000105426 | *PTPRS* |
| ENSG00000088882 | *CPXM1* | ENSG00000073008 | *PVR* |
| ENSG00000130962 | *PRRG1* | ENSG00000148143 | *ZNF462* |
| ENSG00000010438 | *PRSS3* | ENSG00000169213 | *RAB3B* |
| ENSG00000189056 | *RELN* | ENSG00000051180 | *RAD51* |
| ENSG00000175352 | *NRIP3* | ENSG00000166349 | *RAG1* |
| ENSG00000111554 | *MDM1* | ENSG00000114200 | *BCHE* |
| ENSG00000176907 | *C8orf4* | ENSG00000172819 | *RARG* |
| ENSG00000125912 | *NCLN* | ENSG00000113319 | *RASGRF2* |
| ENSG00000100804 | *PSMB5* | ENSG00000032219 | *ARID4A* |
| ENSG00000116774 | *OLFML3* | ENSG00000074527 | *NTN4* |
| ENSG00000099256 | *PRTFDC1* | ENSG00000126950 | *TMEM35A* |
| ENSG00000240065 | *PSMB9* | ENSG00000114115 | *RBP1* |
| ENSG00000104047 | *DTWD1* | ENSG00000171791 | *BCL2* |
| ENSG00000163808 | *KIF15* | ENSG00000049541 | *RFC2* |
| ENSG00000144476 | *ACKR3* | ENSG00000138835 | *RGS3* |
| ENSG00000090097 | *PCBP4* | ENSG00000269900 | *RMRP* |
| ENSG00000175166 | *PSMD2* | ENSG00000283029 | *RN7SL1* |
| ENSG00000047617 | *ANO2* | ENSG00000149201 | *CCDC81* |
| ENSG00000101474 | *APMAP* | ENSG00000106399 | *RPA3* |
| ENSG00000115310 | *RTN4* | ENSG00000167526 | *RPL13* |
| ENSG00000102897 | *LYRM1* | ENSG00000265681 | *RPL17* |
| ENSG00000139946 | *PELI2* | ENSG00000214026 | *MRPL23* |
| ENSG00000128203 | *ASPHD2* | ENSG00000128626 | *MRPS12* |
| ENSG00000124212 | *PTGIS* | ENSG00000164684 | *ZNF704* |
| ENSG00000100626 | *GALNT16* | ENSG00000170889 | *RPS9* |
| ENSG00000120278 | *PLEKHG1* | ENSG00000171848 | *RRM2* |
| ENSG00000186260 | *MKL2* | ENSG00000176697 | *BDNF* |
| ENSG00000168916 | *ZNF608* | ENSG00000197956 | *S100A6* |
| ENSG00000129422 | *MTUS1* | ENSG00000005187 | *ACSM3* |
| ENSG00000138411 | *HECW2* | ENSG00000137266 | *SLC22A23* |
| ENSG00000136383 | *ALPK3* | ENSG00000182492 | *BGN* |
| ENSG00000144959 | *NCEH1* | ENSG00000196876 | *SCN8A* |
| ENSG00000092421 | *SEMA6A* | ENSG00000108691 | *CCL2* |
| ENSG00000138134 | *STAMBPL1* | ENSG00000115884 | *SDC1* |
| ENSG00000110318 | *CEP126* | ENSG00000169439 | *SDC2* |
| ENSG00000137727 | *ARHGAP20* | ENSG00000154864 | *PIEZO2* |
| ENSG00000144583 | *MARCH4* | ENSG00000101194 | *SLC17A9* |
| ENSG00000105559 | *PLEKHA4* | ENSG00000127586 | *CHTF18* |
| ENSG00000115109 | *EPB41L5* | ENSG00000007908 | *SELE* |
| ENSG00000116260 | *QSOX1* | ENSG00000174175 | *SELP* |
| ENSG00000013293 | *SLC7A14* | ENSG00000001617 | *SEMA3F* |
| ENSG00000153707 | *PTPRD* | ENSG00000129595 | *EPB41L4A* |
| ENSG00000142949 | *PTPRF* | ENSG00000123219 | *CENPK* |
| ENSG00000163661 | *PTX3* | ENSG00000169718 | *DUS1L* |
| ENSG00000117569 | *PTBP2* | ENSG00000142910 | *TINAGL1* |
| ENSG00000073008 | *PVR* | ENSG00000109805 | *NCAPG* |
| ENSG00000137767 | *SQRDL* | ENSG00000267313 | *KC6* |
| ENSG00000128805 | *ARHGAP22* | ENSG00000186283 | *TOR3A* |
| ENSG00000082126 | *MPP4* | ENSG00000214944 | *ARHGEF28* |
| ENSG00000128340 | *RAC2* | ENSG00000164736 | *SOX17* |
| ENSG00000144118 | *RALB* | ENSG00000250337 | *LINC01021* |
| ENSG00000077092 | *RARB* | ENSG00000164161 | *HHIP* |
| ENSG00000113319 | *RASGRF2* | ENSG00000143429 | *LOC645166* |
| ENSG00000032219 | *ARID4A* | ENSG00000206432 | *TMEM200C* |
| ENSG00000198258 | *UBL5* | ENSG00000235770 | *LINC00607* |
| ENSG00000122257 | *RBBP6* | ENSG00000176974 | *SHMT1* |
| ENSG00000101773 | *RBBP8* | ENSG00000136205 | *TNS3* |
| ENSG00000102317 | *RBM3* | ENSG00000075420 | *FNDC3B* |
| ENSG00000114115 | *RBP1* | ENSG00000110080 | *ST3GAL4* |
| ENSG00000110092 | *CCND1* | ENSG00000136830 | *FAM129B* |
| ENSG00000171552 | *BCL2L1* | ENSG00000180992 | *MRPL14* |
| ENSG00000116741 | *RGS2* | ENSG00000125901 | *MRPS26* |
| ENSG00000117152 | *RGS4* | ENSG00000136603 | *SKIL* |
| ENSG00000129538 | *RNASE1* | ENSG00000105281 | *SLC1A5* |
| ENSG00000113916 | *BCL6* | ENSG00000125351 | *UPF3B* |
| ENSG00000149201 | *CCDC81* | ENSG00000125378 | *BMP4* |
| ENSG00000123395 | *ATG101* | ENSG00000196632 | *WNK3* |
| ENSG00000156313 | *RPGR* | ENSG00000183598 | *HIST2H3D* |
| ENSG00000164684 | *ZNF704* | ENSG00000153162 | *BMP6* |
| ENSG00000175634 | *RPS6KB2* | ENSG00000189223 | *PAX8-AS1* |
| ENSG00000176697 | *BDNF* | ENSG00000234912 | *SNHG20* |
| ENSG00000134243 | *SORT1* | ENSG00000214814 | *FER1L6* |
| ENSG00000197956 | *S100A6* | ENSG00000173638 | *SLC19A1* |
| ENSG00000182568 | *SATB1* | ENSG00000103254 | *FAM173A* |
| ENSG00000204842 | *ATXN2* | ENSG00000108604 | *SMARCD2* |
| ENSG00000182492 | *BGN* | ENSG00000023608 | *SNAPC1* |
| ENSG00000169432 | *SCN9A* | ENSG00000125835 | *SNRPB* |
| ENSG00000108691 | *CCL2* | ENSG00000172331 | *BPGM* |
| ENSG00000276409 | *CCL14* | ENSG00000152377 | *SPOCK1* |
| ENSG00000124875 | *CXCL6* | ENSG00000163554 | *SPTA1* |
| ENSG00000169439 | *SDC2* | ENSG00000140319 | *SRP14* |
| ENSG00000124145 | *SDC4* | ENSG00000144681 | *STAC* |
| ENSG00000154864 | *PIEZO2* | ENSG00000115415 | *STAT1* |
| ENSG00000132436 | *FIGNL1* | ENSG00000138378 | *STAT4* |
| ENSG00000007908 | *SELE* | ENSG00000252835 | *SCARNA21* |
| ENSG00000188404 | *SELL* | ENSG00000270066 | *SCARNA2* |
| ENSG00000112378 | *PERP* | ENSG00000252481 | *SCARNA13* |
| ENSG00000155307 | *SAMSN1* | ENSG00000251791 | *SCARNA6* |
| ENSG00000135926 | *TMBIM1* | ENSG00000252010 | *SCARNA5* |
| ENSG00000142910 | *TINAGL1* | ENSG00000212464 | *SNORA12* |
| ENSG00000015532 | *XYLT2* | ENSG00000206634 | *SNORA22* |
| ENSG00000115267 | *IFIH1* | ENSG00000238961 | *SNORA47* |
| ENSG00000178980 | *SELENOW* | ENSG00000200354 | *SNORA71D* |
| ENSG00000214765 | *SEPT7P2* | ENSG00000159167 | *STC1* |
| ENSG00000154134 | *ROBO3* | ENSG00000109193 | *SULT1E1* |
| ENSG00000007384 | *RHBDF1* | ENSG00000087586 | *AURKA* |
| ENSG00000164736 | *SOX17* | ENSG00000130303 | *BST2* |
| ENSG00000167693 | *NXN* | ENSG00000067715 | *SYT1* |
| ENSG00000235505 | *LOC643733* | ENSG00000149591 | *TAGLN* |
| ENSG00000118515 | *SGK1* | ENSG00000112592 | *TBP* |
| ENSG00000166922 | *SCG5* | ENSG00000239039 | *SNORD13* |
| ENSG00000143429 | *LOC645166* | ENSG00000212232 | *SNORD17* |
| ENSG00000108854 | *SMURF2* | ENSG00000196628 | *TCF4* |
| ENSG00000136205 | *TNS3* | ENSG00000137310 | *TCF19* |
| ENSG00000127080 | *IPPK* | ENSG00000185339 | *TCN2* |
| ENSG00000075420 | *FNDC3B* | ENSG00000169679 | *BUB1* |
| ENSG00000121749 | *TBC1D15* | ENSG00000156970 | *BUB1B* |
| ENSG00000073849 | *ST6GAL1* | ENSG00000270141 | *TERC* |
| ENSG00000136830 | *FAM129B* | ENSG00000090447 | *TFAP4* |
| ENSG00000163814 | *CDCP1* | ENSG00000072274 | *TFRC* |
| ENSG00000112246 | *SIM1* | ENSG00000092969 | *TGFB2* |
| ENSG00000180992 | *MRPL14* | ENSG00000120708 | *TGFBI* |
| ENSG00000153044 | *CENPH* | ENSG00000106799 | *TGFBR1* |
| ENSG00000165271 | *NOL6* | ENSG00000137801 | *THBS1* |
| ENSG00000125351 | *UPF3B* | ENSG00000100300 | *TSPO* |
| ENSG00000164889 | *SLC4A2* | ENSG00000172009 | *THOP1* |
| ENSG00000214814 | *FER1L6* | ENSG00000035862 | *TIMP2* |
| ENSG00000090020 | *SLC9A1* | ENSG00000167900 | *TK1* |
| ENSG00000064651 | *SLC12A2* | ENSG00000120802 | *TMPO* |
| ENSG00000144136 | *SLC20A1* | ENSG00000184113 | *CLDN5* |
| ENSG00000174640 | *SLCO2A1* | ENSG00000131747 | *TOP2A* |
| ENSG00000080503 | *SMARCA2* | ENSG00000146242 | *TPBG* |
| ENSG00000102010 | *BMX* | ENSG00000111669 | *TPI1* |
| ENSG00000166311 | *SMPD1* | ENSG00000140416 | *TPM1* |
| ENSG00000117143 | *UAP1* | ENSG00000067445 | *TRO* |
| ENSG00000114850 | *SSR3* | ENSG00000106804 | *C5* |
| ENSG00000139618 | *BRCA2* | ENSG00000185561 | *TLCD2* |
| ENSG00000115415 | *STAT1* | ENSG00000176890 | *TYMS* |
| ENSG00000138378 | *STAT4* | ENSG00000213967 | *ZNF726* |
| ENSG00000126561 | *STAT5A* | ENSG00000148154 | *UGCG* |
| ENSG00000252010 | *SCARNA5* | ENSG00000149823 | *VPS51* |
| ENSG00000206634 | *SNORA22* | ENSG00000162692 | *VCAM1* |
| ENSG00000201998 | *SNORA23* | ENSG00000140105 | *WARS* |
| ENSG00000212443 | *SNORA53* | ENSG00000147180 | *ZNF711* |
| ENSG00000152518 | *ZFP36L2* | ENSG00000167232 | *ZNF91* |
| ENSG00000109193 | *SULT1E1* | ENSG00000159840 | *ZYX* |
| ENSG00000109743 | *BST1* | ENSG00000263934 | *SNORD3A* |
| ENSG00000130303 | *BST2* | ENSG00000144063 | *MALL* |
| ENSG00000149591 | *TAGLN* | ENSG00000214706 | *IFRD2* |
| ENSG00000197780 | *TAF13* | ENSG00000075785 | *RAB7A* |
| ENSG00000204219 | *TCEA3* | ENSG00000169282 | *KCNAB1* |
| ENSG00000212232 | *SNORD17* | ENSG00000089723 | *OTUB2* |
| ENSG00000005436 | *GCFC2* | ENSG00000173621 | *LRFN4* |
| ENSG00000169131 | *ZNF354A* | ENSG00000100162 | *CENPM* |
| ENSG00000145022 | *TCTA* | ENSG00000110104 | *CCDC86* |
| ENSG00000003436 | *TFPI* | ENSG00000116455 | *WDR77* |
| ENSG00000072274 | *TFRC* | ENSG00000111670 | *GNPTAB* |
| ENSG00000042832 | *TG* | ENSG00000142634 | *EFHD2* |
| ENSG00000092969 | *TGFB2* | ENSG00000131652 | *THOC6* |
| ENSG00000120708 | *TGFBI* | ENSG00000111058 | *ACSS3* |
| ENSG00000069702 | *TGFBR3* | ENSG00000119900 | *OGFRL1* |
| ENSG00000100300 | *TSPO* | ENSG00000196159 | *FAT4* |
| ENSG00000104067 | *TJP1* | ENSG00000129680 | *MAP7D3* |
| ENSG00000065717 | *TLE2* | ENSG00000173281 | *PPP1R3B* |
| ENSG00000028137 | *TNFRSF1B* | ENSG00000100379 | *KCTD17* |
| ENSG00000159403 | *C1R* | ENSG00000063241 | *ISOC2* |
| ENSG00000131747 | *TOP2A* | ENSG00000175471 | *MCTP1* |
| ENSG00000146242 | *TPBG* | ENSG00000091656 | *ZFHX4* |
| ENSG00000076554 | *TPD52* | ENSG00000165959 | *CLMN* |
| ENSG00000140416 | *TPM1* | ENSG00000175213 | *ZNF408* |
| ENSG00000198467 | *TPM2* | ENSG00000171241 | *SHCBP1* |
| ENSG00000067445 | *TRO* | ENSG00000116771 | *AGMAT* |
| ENSG00000155657 | *TTN* | ENSG00000150636 | *CCDC102B* |
| ENSG00000137267 | *TUBB2A* | ENSG00000136122 | *BORA* |
| ENSG00000143367 | *TUFT1* | ENSG00000187720 | *THSD4* |
| ENSG00000117586 | *TNFSF4* | ENSG00000135362 | *PRR5L* |
| ENSG00000226053 | *LOC729987* | ENSG00000133142 | *TCEAL4* |
| ENSG00000148154 | *UGCG* | ENSG00000092470 | *WDR76* |
| ENSG00000183255 | *PTTG1IP* | ENSG00000149636 | *DSN1* |
| ENSG00000189180 | *ZNF33A* | ENSG00000122786 | *CALD1* |
| ENSG00000202048 | *SNORD114-20* | ENSG00000137872 | *SEMA6D* |
| ENSG00000063180 | *CA11* | ENSG00000140451 | *PIF1* |
| ENSG00000109906 | *ZBTB16* | ENSG00000138759 | *FRAS1* |
| ENSG00000147394 | *ZNF185* | ENSG00000122490 | *PQLC1* |
| ENSG00000149050 | *ZNF214* | ENSG00000278259 | *MYO19* |
| ENSG00000163879 | *DNALI1* | ENSG00000119242 | *CCDC92* |
| ENSG00000162511 | *LAPTM5* | ENSG00000156675 | *RAB11FIP1* |
| ENSG00000263934 | *SNORD3A* | ENSG00000046889 | *PREX2* |
| ENSG00000115275 | *MOGS* | ENSG00000138336 | *TET1* |
| ENSG00000167552 | *TUBA1A* | ENSG00000177192 | *PUS1* |
| ENSG00000121966 | *CXCR4* | ENSG00000111981 | *ULBP1* |
| ENSG00000169282 | *KCNAB1* | ENSG00000197646 | *PDCD1LG2* |
| ENSG00000185803 | *SLC52A2* | ENSG00000221963 | *APOL6* |
| ENSG00000111058 | *ACSS3* | ENSG00000100836 | *PABPN1* |
| ENSG00000119900 | *OGFRL1* | ENSG00000158555 | *GDPD5* |
| ENSG00000196159 | *FAT4* | ENSG00000101447 | *FAM83D* |
| ENSG00000180801 | *ARSJ* | ENSG00000167513 | *CDT1* |
| ENSG00000187240 | *DYNC2H1* | ENSG00000178605 | *GTPBP6* |
| ENSG00000128915 | *ICE2* | ENSG00000169249 | *ZRSR2* |
| ENSG00000196449 | *YRDC* | ENSG00000276180 | *HIST1H4I* |
| ENSG00000119514 | *GALNT12* | ENSG00000093009 | *CDC45* |
| ENSG00000130309 | *COLGALT1* | ENSG00000196747 | *HIST1H2AI* |
| ENSG00000198517 | *MAFK* | ENSG00000275221 | *HIST1H2AK* |
| ENSG00000175471 | *MCTP1* | ENSG00000276368 | *HIST1H2AJ* |
| ENSG00000132321 | *IQCA1* | ENSG00000276903 | *HIST1H2AL* |
| ENSG00000175213 | *ZNF408* | ENSG00000278463 | *HIST1H2AB* |
| ENSG00000105825 | *TFPI2* | ENSG00000185130 | *HIST1H2BL* |
| ENSG00000105792 | *CFAP69* | ENSG00000274290 | *HIST1H2BE* |
| ENSG00000187720 | *THSD4* | ENSG00000275713 | *HIST1H2BH* |
| ENSG00000104723 | *TUSC3* | ENSG00000278588 | *HIST1H2BI* |
| ENSG00000133142 | *TCEAL4* | ENSG00000111665 | *CDCA3* |
| ENSG00000099219 | *ERMP1* | ENSG00000213347 | *MXD3* |
| ENSG00000121895 | *TMEM156* | ENSG00000129932 | *DOHH* |
| ENSG00000266714 | *MYO15B* | ENSG00000274641 | *HIST1H2BO* |
| ENSG00000162174 | *ASRGL1* | ENSG00000275714 | *HIST1H3A* |
| ENSG00000096872 | *IFT74* | ENSG00000278272 | *HIST1H3C* |
| ENSG00000046889 | *PREX2* | ENSG00000275379 | *HIST1H3I* |
| ENSG00000005238 | *FAM214B* | ENSG00000273983 | *HIST1H3G* |
| ENSG00000107611 | *CUBN* | ENSG00000197153 | *HIST1H3J* |
| ENSG00000165113 | *GKAP1* | ENSG00000278828 | *HIST1H3H* |
| ENSG00000114107 | *CEP70* | ENSG00000274267 | *HIST1H3B* |
| ENSG00000101152 | *DNAJC5* | ENSG00000278637 | *HIST1H4A* |
| ENSG00000196812 | *ZSCAN16* | ENSG00000126453 | *BCL2L12* |
| ENSG00000197646 | *PDCD1LG2* | ENSG00000277157 | *HIST1H4D* |
| ENSG00000128284 | *APOL3* | ENSG00000273542 | *HIST1H4K* |
| ENSG00000158270 | *COLEC12* | ENSG00000197238 | *HIST1H4J* |
| ENSG00000205683 | *DPF3* | ENSG00000197061 | *HIST1H4C* |
| ENSG00000140350 | *ANP32A* | ENSG00000158406 | *HIST1H4H* |
| ENSG00000116667 | *C1orf21* | ENSG00000276966 | *HIST1H4E* |
| ENSG00000239264 | *TXNDC5* | ENSG00000275126 | *HIST1H4L* |
| ENSG00000178878 | *APOLD1* | ENSG00000149781 | *FERMT3* |
| ENSG00000124749 | *COL21A1* | ENSG00000105447 | *GRWD1* |
| ENSG00000143401 | *ANP32E* | ENSG00000126391 | *FRMD8* |
| ENSG00000140941 | *MAP1LC3B* | ENSG00000160072 | *ATAD3B* |
| ENSG00000062716 | *VMP1* | ENSG00000143341 | *HMCN1* |
| ENSG00000145349 | *CAMK2D* | ENSG00000130307 | *USHBP1* |
| ENSG00000178922 | *HYI* | ENSG00000144354 | *CDCA7* |
| ENSG00000184260 | *HIST2H2AC* | ENSG00000177602 | *GSG2* |
| ENSG00000275713 | *HIST1H2BH* | ENSG00000136492 | *BRIP1* |
| ENSG00000130300 | *PLVAP* | ENSG00000124074 | *ENKD1* |
| ENSG00000126878 | *AIF1L* | ENSG00000064012 | *CASP8* |
| ENSG00000277157 | *HIST1H4D* | ENSG00000140853 | *NLRC5* |
| ENSG00000149781 | *FERMT3* | ENSG00000169604 | *ANTXR1* |
| ENSG00000136169 | *SETDB2* | ENSG00000242125 | *SNHG3* |
| ENSG00000133687 | *TMTC1* | ENSG00000118473 | *SGIP1* |
| ENSG00000119632 | *IFI27L2* | ENSG00000183011 | *NAA38* |
| ENSG00000136492 | *BRIP1* | ENSG00000256053 | *APOPT1* |
| ENSG00000158710 | *TAGLN2* | ENSG00000184384 | *MAML2* |
| ENSG00000126653 | *NSRP1* | ENSG00000145794 | *MEGF10* |
| ENSG00000115363 | *EVA1A* | ENSG00000154556 | *SORBS2* |
| ENSG00000169604 | *ANTXR1* | ENSG00000183615 | *FAM167B* |
| ENSG00000242125 | *SNHG3* | ENSG00000161551 | *ZNF577* |
| ENSG00000121851 | *POLR3GL* | ENSG00000167747 | *C19orf48* |
| ENSG00000186812 | *ZNF397* | ENSG00000163702 | *IL17RC* |
| ENSG00000168101 | *NUDT16L1* | ENSG00000176619 | *LMNB2* |
| ENSG00000122986 | *HVCN1* | ENSG00000185904 | *LINC00839* |
| ENSG00000168497 | *SDPR* | ENSG00000099910 | *KLHL22* |
| ENSG00000198286 | *CARD11* | ENSG00000135637 | *CCDC142* |
| ENSG00000166432 | *ZMAT1* | ENSG00000092208 | *GEMIN2* |
| ENSG00000176014 | *TUBB6* | ENSG00000146374 | *RSPO3* |
| ENSG00000167553 | *TUBA1C* | ENSG00000164099 | *PRSS12* |
| ENSG00000223749 | *MIR503HG* | ENSG00000102802 | *MEDAG* |
| ENSG00000166387 | *PPFIBP2* | ENSG00000233016 | *SNHG7* |
| ENSG00000128849 | *CGNL1* | ENSG00000196507 | *TCEAL3* |
| ENSG00000129474 | *AJUBA* | ENSG00000274997 | *HIST1H2AH* |
| ENSG00000149150 | *SLC43A1* | ENSG00000197903 | *HIST1H2BK* |
| ENSG00000196507 | *TCEAL3* | ENSG00000180543 | *TSPYL5* |
| ENSG00000144283 | *PKP4* | ENSG00000105499 | *PLA2G4C* |
| ENSG00000108797 | *CNTNAP1* | ENSG00000162407 | *PLPP3* |
| ENSG00000171617 | *ENC1* | ENSG00000113739 | *STC2* |
| ENSG00000143127 | *ITGA10* | ENSG00000138735 | *PDE5A* |
| ENSG00000170915 | *PAQR8* | ENSG00000118640 | *VAMP8* |
| ENSG00000143013 | *LMO4* | ENSG00000068001 | *HYAL2* |
| ENSG00000087842 | *PIR* | ENSG00000159228 | *CBR1* |
| ENSG00000130589 | *HELZ2* | ENSG00000159231 | *CBR3* |
| ENSG00000133739 | *LRRCC1* | ENSG00000143537 | *ADAM15* |
| ENSG00000150764 | *DIXDC1* | ENSG00000157873 | *TNFRSF14* |
| ENSG00000134107 | *BHLHE40* | ENSG00000128918 | *ALDH1A2* |
| ENSG00000105499 | *PLA2G4C* | ENSG00000138074 | *SLC5A6* |
| ENSG00000162407 | *PLPP3* | ENSG00000106052 | *TAX1BP1* |
| ENSG00000079102 | *RUNX1T1* | ENSG00000145386 | *CCNA2* |
| ENSG00000196139 | *AKR1C3* | ENSG00000133101 | *CCNA1* |
| ENSG00000117533 | *VAMP4* | ENSG00000134057 | *CCNB1* |
| ENSG00000162734 | *PEA15* | ENSG00000118971 | *CCND2* |
| ENSG00000068001 | *HYAL2* | ENSG00000277775 | *HIST1H3F* |
| ENSG00000121858 | *TNFSF10* | ENSG00000196787 | *HIST1H2AG* |
| ENSG00000143537 | *ADAM15* | ENSG00000124635 | *HIST1H2BJ* |
| ENSG00000168615 | *ADAM9* | ENSG00000184897 | *H1FX* |
| ENSG00000141655 | *TNFRSF11A* | ENSG00000168010 | *ATG16L2* |
| ENSG00000115604 | *IL18R1* | ENSG00000119333 | *WDR34* |
| ENSG00000196468 | *FGF16* | ENSG00000162063 | *CCNF* |
| ENSG00000137563 | *GGH* | ENSG00000120337 | *TNFSF18* |
| ENSG00000171388 | *APLN* | ENSG00000186193 | *SAPCD2* |
| ENSG00000176170 | *SPHK1* | ENSG00000144824 | *PHLDB2* |
| ENSG00000133101 | *CCNA1* | ENSG00000238227 | *C9orf69* |
| ENSG00000118971 | *CCND2* | ENSG00000171004 | *HS6ST2* |
| ENSG00000067798 | *NAV3* | ENSG00000003096 | *KLHL13* |
| ENSG00000168010 | *ATG16L2* | ENSG00000110711 | *AIP* |
| ENSG00000172578 | *KLHL6* | ENSG00000141295 | *SCRN2* |
| ENSG00000113732 | *ATP6V0E1* | ENSG00000198901 | *PRC1* |
| ENSG00000120337 | *TNFSF18* | ENSG00000129691 | *ASH2L* |
| ENSG00000127533 | *F2RL3* | ENSG00000163347 | *CLDN1* |
| ENSG00000013588 | *GPRC5A* | ENSG00000137033 | *IL33* |
| ENSG00000139597 | *N4BP2L1* | ENSG00000168679 | *SLC16A4* |
| ENSG00000129691 | *ASH2L* | ENSG00000157456 | *CCNB2* |
| ENSG00000134873 | *CLDN10* | ENSG00000114767 | *RRP9* |
| ENSG00000163347 | *CLDN1* | ENSG00000174371 | *EXO1* |
| ENSG00000162595 | *DIRAS3* | ENSG00000162614 | *NEXN* |
| ENSG00000169744 | *LDB2* | ENSG00000179820 | *MYADM* |
| ENSG00000258890 | *CEP95* | ENSG00000120334 | *CENPL* |
| ENSG00000134668 | *SPOCD1* | ENSG00000073803 | *MAP3K13* |
| ENSG00000137033 | *IL33* | ENSG00000198774 | *RASSF9* |
| ENSG00000109881 | *CCDC34* | ENSG00000133083 | *DCLK1* |
| ENSG00000141526 | *SLC16A3* | ENSG00000178999 | *AURKB* |
| ENSG00000107438 | *PDLIM1* | ENSG00000171451 | *DSEL* |
| ENSG00000165915 | *SLC39A13* | ENSG00000164611 | *PTTG1* |
| ENSG00000187051 | *RPS19BP1* | ENSG00000183691 | *NOG* |
| ENSG00000154359 | *LONRF1* | ENSG00000128487 | *SPECC1* |
| ENSG00000115602 | *IL1RL1* | ENSG00000017483 | *SLC38A5* |
| ENSG00000198774 | *RASSF9* | ENSG00000181218 | *HIST3H2A* |
| ENSG00000262919 | *FAM58A* | ENSG00000071539 | *TRIP13* |
| ENSG00000167874 | *TMEM88* | ENSG00000105676 | *ARMC6* |
| ENSG00000151276 | *MAGI1* | ENSG00000198542 | *ITGBL1* |
| ENSG00000008517 | *IL32* | ENSG00000145348 | *TBCK* |
| ENSG00000183691 | *NOG* | ENSG00000101670 | *LIPG* |
| ENSG00000100784 | *RPS6KA5* | ENSG00000134824 | *FADS2* |
| ENSG00000196923 | *PDLIM7* | ENSG00000200913 | *SNORD46* |
| ENSG00000081320 | *STK17B* | ENSG00000099337 | *KCNK6* |
| ENSG00000010278 | *CD9* | ENSG00000118777 | *ABCG2* |
| ENSG00000178031 | *ADAMTSL1* | ENSG00000172071 | *EIF2AK3* |
| ENSG00000205038 | *PKHD1L1* | ENSG00000129675 | *ARHGEF6* |
| ENSG00000134986 | *NREP* | ENSG00000158859 | *ADAMTS4* |
| ENSG00000198542 | *ITGBL1* | ENSG00000154734 | *ADAMTS1* |
| ENSG00000156787 | *TBC1D31* | ENSG00000112208 | *BAG2* |
| ENSG00000138398 | *PPIG* | ENSG00000130755 | *GMFG* |
| ENSG00000101670 | *LIPG* | ENSG00000138433 | *CIR1* |
| ENSG00000152465 | *NMT2* | ENSG00000146592 | *CREB5* |
| ENSG00000110031 | *LPXN* | ENSG00000131016 | *AKAP12* |
| ENSG00000172057 | *ORMDL3* | ENSG00000050344 | *NFE2L3* |
| ENSG00000118777 | *ABCG2* | ENSG00000174791 | *RIN1* |
| ENSG00000135218 | *CD36* | ENSG00000104728 | *ARHGEF10* |
| ENSG00000179841 | *AKAP5* | ENSG00000100034 | *PPM1F* |
| ENSG00000050438 | *SLC4A8* | ENSG00000110848 | *CD69* |
| ENSG00000154734 | *ADAMTS1* | ENSG00000135476 | *ESPL1* |
| ENSG00000130513 | *GDF15* | ENSG00000170160 | *CCDC144A* |
| ENSG00000138185 | *ENTPD1* | ENSG00000170381 | *SEMA3E* |
| ENSG00000115129 | *TP53I3* | ENSG00000138593 | *SECISBP2L* |
| ENSG00000138182 | *KIF20B* | ENSG00000048052 | *HDAC9* |
| ENSG00000146592 | *CREB5* | ENSG00000007312 | *CD79B* |
| ENSG00000131016 | *AKAP12* | ENSG00000166803 | *KIAA0101* |
| ENSG00000026508 | *CD44* | ENSG00000126787 | *DLGAP5* |
| ENSG00000050344 | *NFE2L3* | ENSG00000184867 | *ARMCX2* |
| ENSG00000187608 | *ISG15* | ENSG00000198826 | *ARHGAP11A* |
| ENSG00000135968 | *GCC2* | ENSG00000170312 | *CDK1* |
| ENSG00000085063 | *CD59* | ENSG00000165304 | *MELK* |
| ENSG00000158417 | *EIF5B* | ENSG00000101003 | *GINS1* |
| ENSG00000135404 | *CD63* | ENSG00000094804 | *CDC6* |
| ENSG00000106443 | *PHF14* | ENSG00000159164 | *SV2A* |
| ENSG00000110848 | *CD69* | ENSG00000117399 | *CDC20* |
| ENSG00000065308 | *TRAM2* | ENSG00000164045 | *CDC25A* |
| ENSG00000166037 | *CEP57* | ENSG00000101224 | *CDC25B* |
| ENSG00000048052 | *HDAC9* | ENSG00000158402 | *CDC25C* |
| ENSG00000110651 | *CD81* | ENSG00000181634 | *TNFSF15* |
| ENSG00000158691 | *ZSCAN12* | ENSG00000100918 | *REC8* |
| ENSG00000130052 | *STARD8* | ENSG00000231711 | NA |
| ENSG00000177697 | *CD151* | ENSG00000257732 | NA |
| ENSG00000198853 | *RUSC2* | ENSG00000259001 | NA |
| ENSG00000065609 | *SNAP91* | ENSG00000266037 | NA |
| ENSG00000159164 | *SV2A* | ENSG00000233452 | NA |
| ENSG00000162413 | *KLHL21* | ENSG00000132967 | NA |
| ENSG00000242802 | *AP5Z1* | ENSG00000261269 | NA |
| ENSG00000241399 | *CD302* | ENSG00000261295 | NA |
| ENSG00000013364 | *MVP* | ENSG00000263426 | NA |
| ENSG00000181634 | *TNFSF15* | ENSG00000263740 | NA |
| ENSG00000100918 | *REC8* | ENSG00000280079 | NA |
| ENSG00000118412 | *CASP8AP2* | ENSG00000281508 | NA |
| ENSG00000232533 | NA | ENSG00000200087 | NA |
| ENSG00000233117 | NA | ENSG00000197083 | NA |
| ENSG00000260604 | NA | ENSG00000200312 | NA |
| ENSG00000233452 | NA | ENSG00000220785 | NA |
| ENSG00000171889 | NA | ENSG00000233117 | NA |
| ENSG00000140181 | NA | ENSG00000175746 | NA |
| ENSG00000279095 | NA | ENSG00000267325 | NA |
| ENSG00000235385 | NA | ENSG00000222489 | NA |
| ENSG00000266037 | NA | ENSG00000124529 | NA |
| ENSG00000257605 | NA | ENSG00000223612 | NA |
| ENSG00000248187 | NA | ENSG00000229855 | NA |
| ENSG00000228742 | NA | ENSG00000244642 | NA |
| ENSG00000175746 | NA | ENSG00000254635 | NA |
| ENSG00000273038 | NA | ENSG00000210082 | NA |
| ENSG00000253177 | NA | ENSG00000226476 | NA |
| ENSG00000225840 | NA | ENSG00000226702 | NA |
| ENSG00000276107 | NA | ENSG00000226958 | NA |
| ENSG00000263426 | NA | ENSG00000265150 | NA |
| ENSG00000198327 | NA | ENSG00000197927 | NA |
| ENSG00000226958 | NA | ENSG00000214176 | NA |
| ENSG00000244398 | NA | ENSG00000205664 | NA |
| ENSG00000282057 | NA | ENSG00000228495 | NA |
| ENSG00000259345 | NA | ENSG00000276107 | NA |
| ENSG00000257732 | NA | ENSG00000198327 | NA |
| ENSG00000228495 | NA | ENSG00000276232 | NA |
| ENSG00000230257 | NA | ENSG00000140181 | NA |
| ENSG00000215386 | NA | ENSG00000237039 | NA |
| ENSG00000231991 | NA | ENSG00000230606 | NA |
| ENSG00000207344 | NA | ENSG00000197846 | NA |
| ENSG00000168405 | NA | ENSG00000237973 | NA |
| ENSG00000226476 | NA | ENSG00000260604 | NA |
| ENSG00000269378 | NA | ENSG00000239437 | NA |
| ENSG00000165121 | NA | ENSG00000235385 | NA |
| ENSG00000231503 | NA | ENSG00000248187 | NA |
