# Supplementary material for: Light modulation ameliorates expression of circadian genes and disease progression in spinal muscular atrophy mice
Source: Hum Mol Genet. 2018 Aug 14;27(20):3582–97. doi: 10.1093/hmg/ddy249 (PMC6168969; doi:10.1093/hmg/ddy249)
Supplement: Supplementary Data [file ddy249_supp.zip › Karjosukarso et al - HMG-2018-D-00441_S5 Table.docx]

| **S5 Table Primers list** | |
| --- | --- |
|  |  |
| **Target** | **Sequence** |
| qPCR_*LYVE1*_Forward | TTTGGAAGGTTCCAGTGAGC |
| qPCR_*LYVE1*_Reverse | GAATATGGGATCTTTGGTGGTG |
| qPCR_*HMOX1*_Forward | CTTTCAGAAGGGCCAGGTG |
| qPCR_*HMOX1*_Reverse | GTAGACAGGGGCGAAGACTG |
| qPCR_*CLDN5*_Forward | CCTTCCTGGACCACAACATC |
| qPCR_*CLDN5*_Reverse | ACCGAGTCGTACACTTTGCAC |
| qPCR_*ALDH1A2*_Forward | ATGATATGCGGATTGCCAAG |
| qPCR_*ALDH1A2*_Reverse | CAGCTGCTACGAGTCCAAAG |
| qPCR_*KHDRBS3*_Forward | TGAAGCTGGGACAGAAAGTG |
| qPCR_*KHDRBS3*_Reverse | AACCTTTCCCAAGGATGGAC |
| qPCR_*FILIP1L*_Forward | AACGCTGGTATCATGGCTGAA |
| qPCR_*FILIP1L*_Reverse | ATCTCTTGCACTGCTCCTCCATT |
| qPCR_*COL8A1*_Forward | TGGCAAAGAGTATCCACACC |
| qPCR_*COL8A1*_Reverse | TTGTTCCCCTCGTAAACTGG |
| qPCR_*CTGF*_Forward | GCAGGCTAGAGAAGCAGAGC |
| qPCR_*CTGF*_Reverse | TGGAGATTTTGGGAGTACGG |
| qPCR_*EDN1*_Forward | ACTTCTGCCACCTGGACATC |
| qPCR_*EDN1*_Reverse | GGCATCTATTTTCACGGTCTG |
| qPCR_*ANGPT2*_Forward | AGGGACAAACCTGTTGAACC |
| qPCR_*ANGPT2*_Reverse | TTGTCGAGAGGGAGTGTTCC |
| qPCR_*ITGA4*_Forward | ACCTCAATGCAGATGGCTTC |
| qPCR_*ITGA4*_Reverse | ACGAGGTTTGTTTCCATTGC |
| qPCR_*MMP2*_Forward | GAGAAGGATGGCAAGTACGG |
| qPCR_*MMP2*_Reverse | CATAGGATGTGCCCTGGAAG |
| qPCR_*PLSCR4*_Forward | ACAGCCTGCAGGTGAAATG |
| qPCR_*PLSCR4*_Reverse | TGGGTAGCCAGTAGGTGGAG |
| qPCR_*TGFB2*_Forward | AGCCAGAGTGCCTGAACAAC |
| qPCR_*TGFB2*_Reverse | ACATCGAAGGAGAGCCATTC |
| qPCR_*VCAM1*_Forward | GGCAGGCTGTAAAAGAATTGC |
| qPCR_*VCAM1*_Reverse | TTCTTGCAGCTTTGTGGATG |
| qPCR_*GUSB*_Forward | AGAGTGGTGCTGAGGATTGG |
| qPCR_*GUSB*_Reverse | CCCTCATGCTCTAGCGTGTC |
| gDNA_*ZNF408*_Forward | CCTGGCCAAGAAGTTACACAG |
| gDNA_*ZNF408*_Reverse | TTTCTCCTGTATGGAGCCTCA |
| qPCR_*ZNF408*_Forward | GAGGAGTCTGCCTCCAAGG |
| qPCR_*ZNF408*_Reverse | CCAGCCAGAACTCTGCTCAC |
| qPCR_*HA-ZNF408*_Forward | TTACGATGTACCGGATTACGC |
| qPCR_*HA-ZNF408*_Reverse | CGGAAGGGTTCCATCCTAAG |
