# Supplementary figures and images for: Light modulation ameliorates expression of circadian genes and disease progression in spinal muscular atrophy mice
Source: Hum Mol Genet. 2018 Aug 14;27(20):3582–97. doi: 10.1093/hmg/ddy249 (PMC6168969; doi:10.1093/hmg/ddy249)

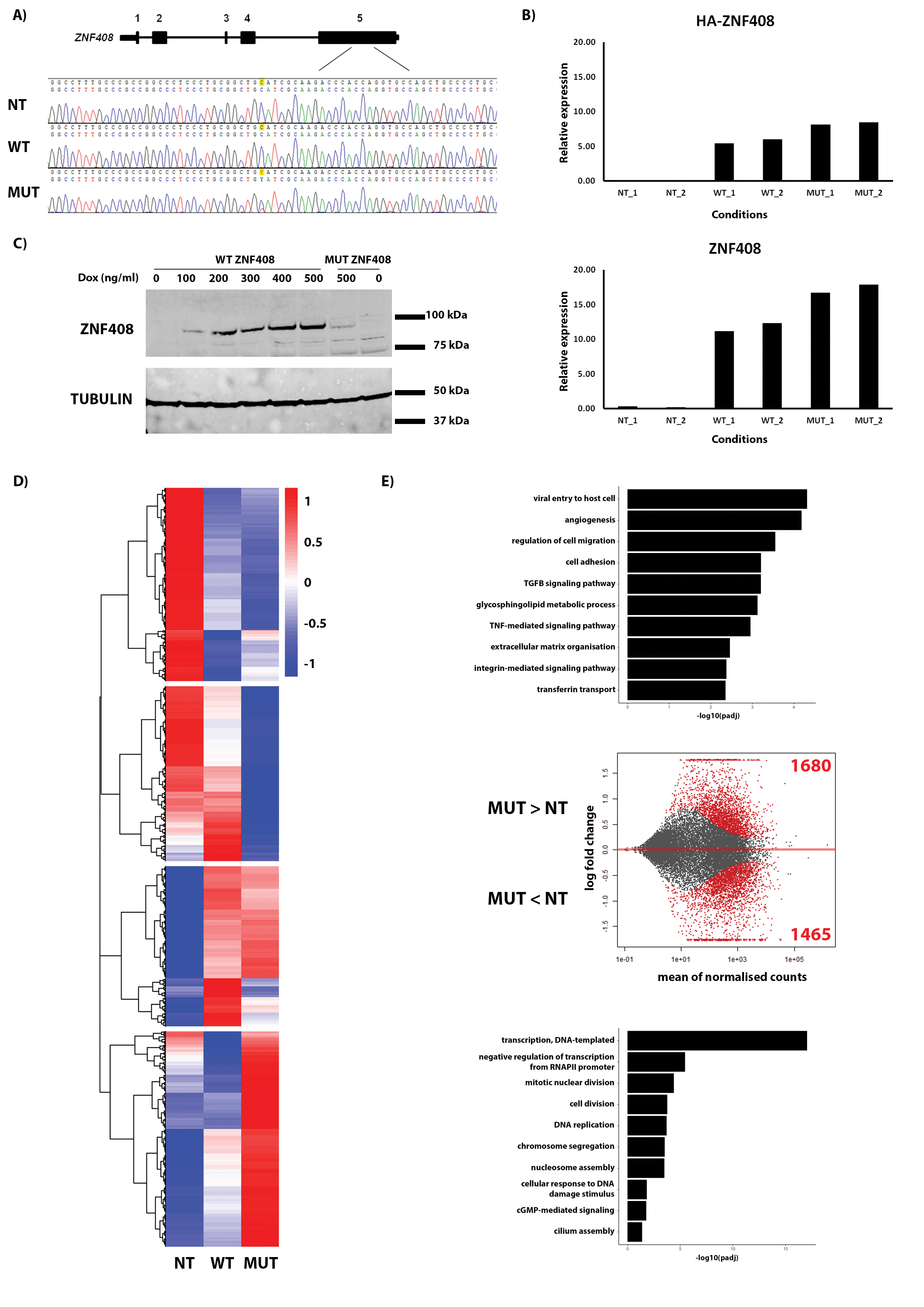

Supplement: Supplementary Data [file ddy249_supp.zip › S1_Fig.tif]

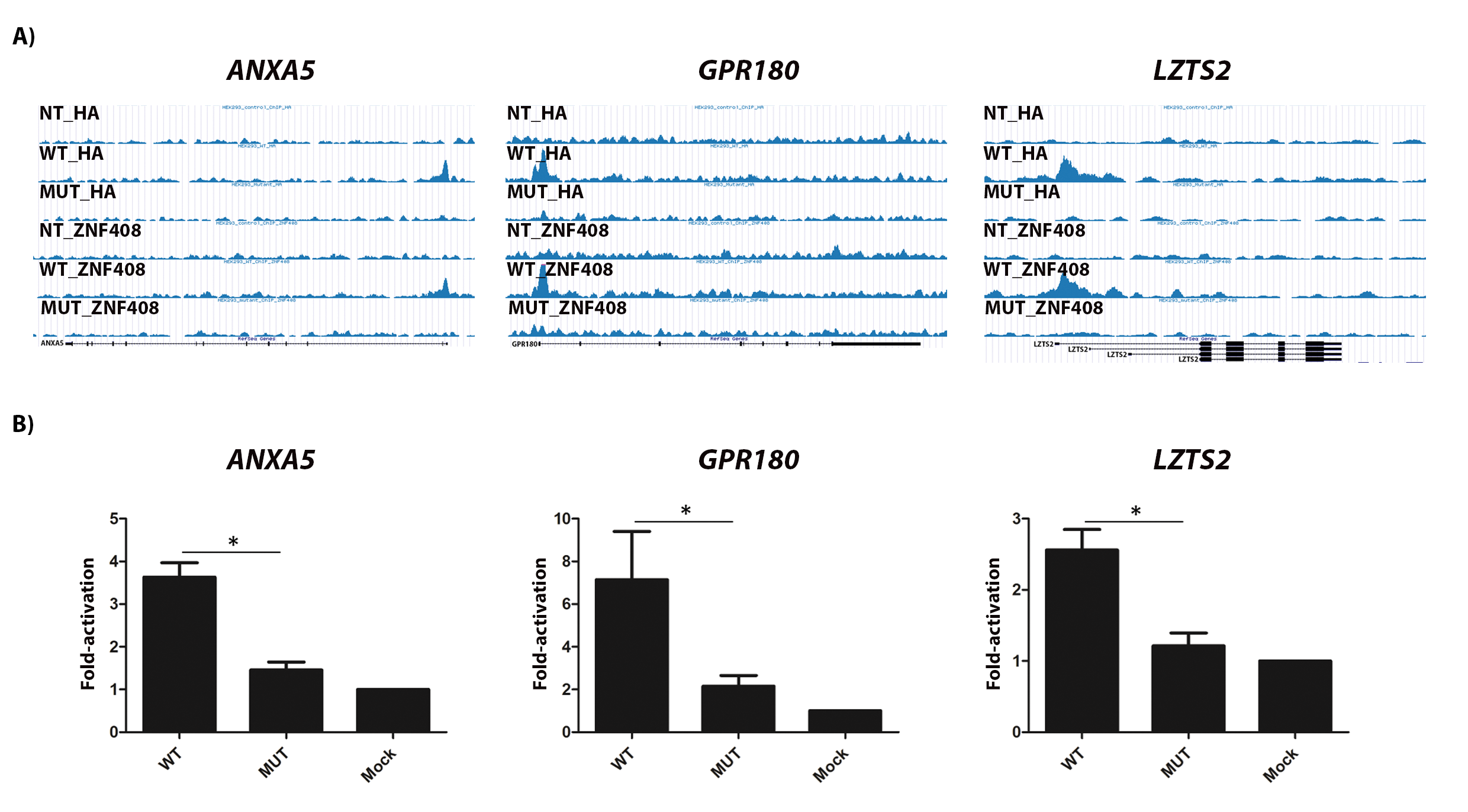

Supplement: Supplementary Data [file ddy249_supp.zip › S3_Fig.tif]

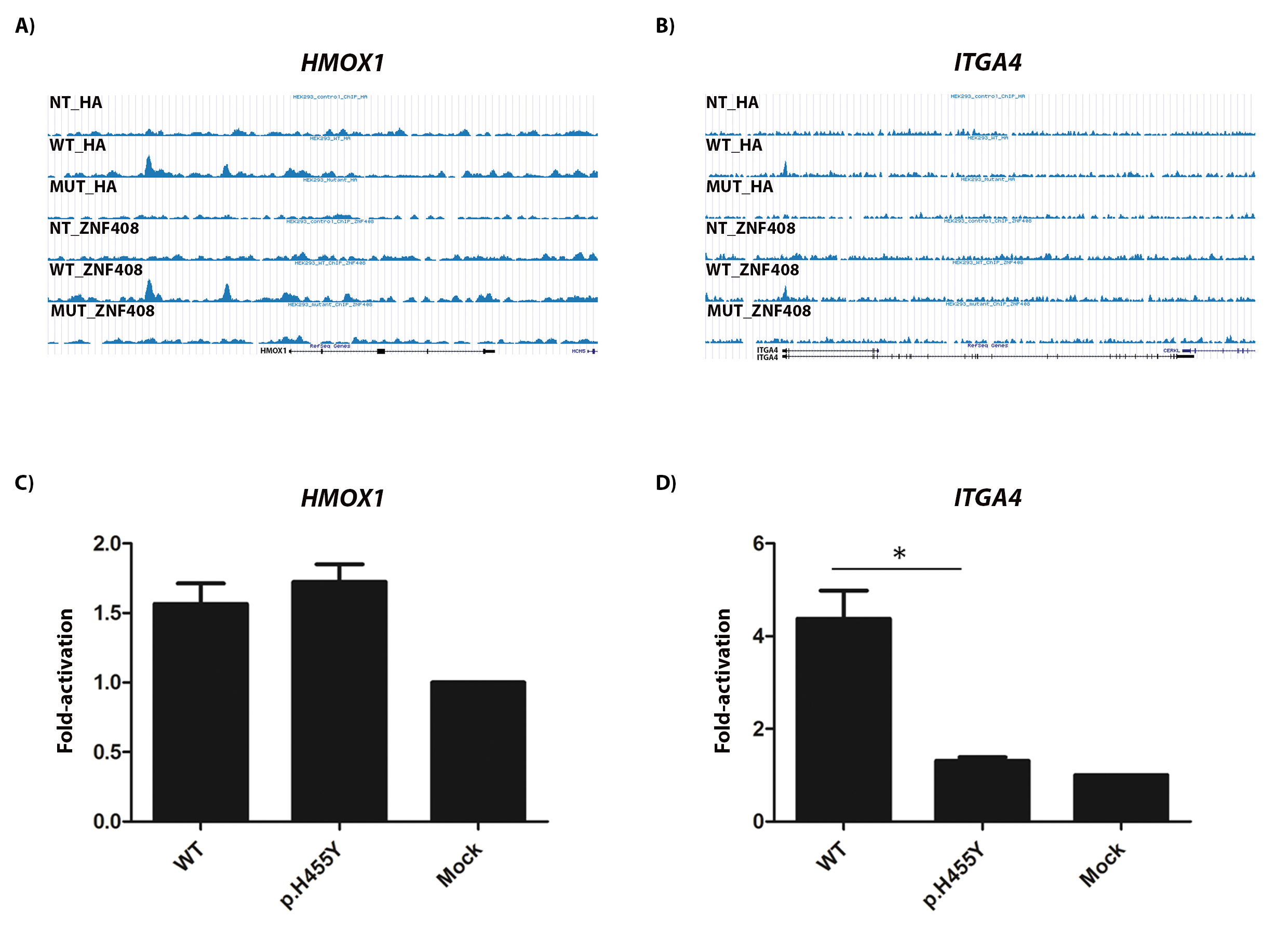

Supplement: Supplementary Data [file ddy249_supp.zip › S4_Fig.tif]

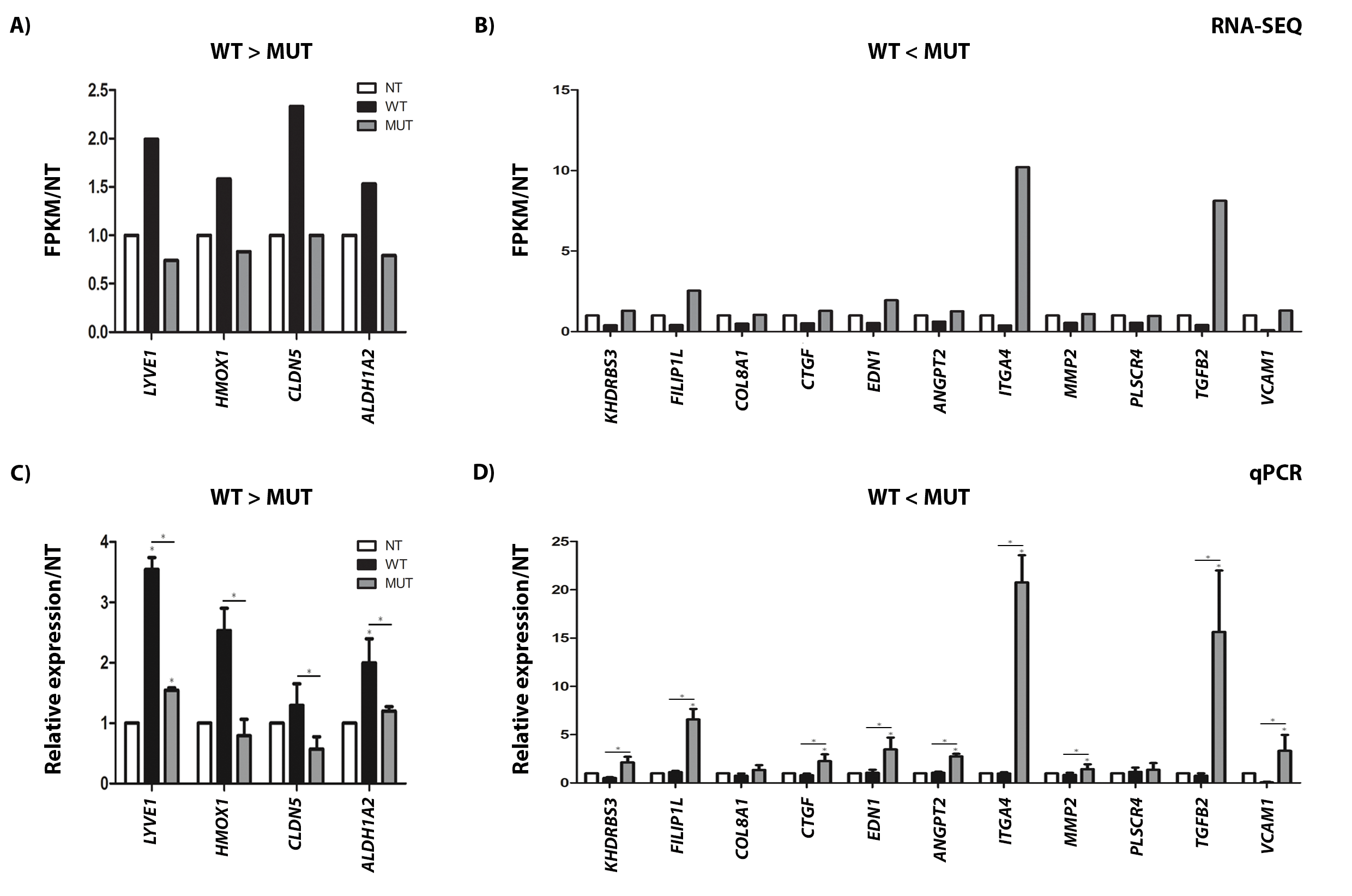

Supplement: Supplementary Data [file ddy249_supp.zip › S2_Fig.tif]
